# 3D-QSAR studies on Maslinic acid analogs for Anticancer activity against Breast Cancer cell line MCF-7

**DOI:** 10.1038/s41598-017-06131-0

**Published:** 2017-07-20

**Authors:** Sarfaraz Alam, Feroz Khan

**Affiliations:** 1 Metabolic & Structural Biology Dept., CSIR-Central Institute of Medicinal & Aromatic Plants, P.O.-CIMAP, Lucknow, 226015 (U.P.) India; 2 Academy of Scientific & Innovative Research (AcSIR), CSIR-CIMAP Campus, Lucknow, 226015 (U.P.) India; 3 Present Address: Skaggs School of Pharmacy & Pharmaceutical Sciences, University of California San Diego (UCSD), 9500 Gilman Drive, La Jolla, San Diego, CA 92093 USA

## Abstract

Global prevalence of breast cancer and its rising frequency makes it a key area of research in drug discovery programs. The research article describes the development of field based 3D-QSAR model based on human breast cancer cell line MCF7 *in vitro* anticancer activity, which defines the molecular level understanding and regions of structure-activity relationship for triterpene maslinic acid and its analogs. The key features such as average shape, hydrophobic regions and electrostatic patterns of active compounds were mined and mapped to virtually screen potential analogs. Then, field points based descriptors were used to develop a 3D-QSAR model by aligning known active compounds onto identified pharmacophore template. The derived LOO validated PLS regression QSAR model showed acceptable r^2^ 0.92 and q^2^ 0.75. After screening through Lipinski’s rule of five filter for oral bioavailability, ADMET risk filter for drug like features, and synthetic accessibility for chemical synthesis, out of 593 hits, 39 were left top hits. Docking screening was performed through identified potential targets namely, AKR1B10, NR3C1, PTGS2, and HER2. Finally, compound P-902 was identified as best hit. This study, would be of great help in lead identification and optimization for early drug discovery.

## Introduction

Breast cancer is the most common cancer among women worldwide. It accounts for nearly 1 in 3 cancers diagnosed in United States women and accounts for 27% of all cancers in Indian women. Nearly 1.7 million new cases diagnosed in 2012^1^. This constitutes about 12% of all new cancer cases and 25% of all cancers in women and supposed to be the leading cause of morbidity and mortality in both pre- and post-menopausal women^2^. In India, cervical cancer has replaced by breast cancer, as the foremost cause of cancer deaths among women. This growing incidence of breast cancer and developing drug resistance to existing anticancer drugs force researchers worldwide to develop new medications in a speedier way^3^. This requirement can be fulfilled by using structure-based drug designing approach in lead identification and optimization, which emerged as a powerful tool to enhance the drug discovery processes^4^. Besides, several researchers explored the potential of plant molecules against cancer such as, indoles, isoflavones, and resveratrol *etc*. Natural plant products serve as an excellent source for the discovery and development of modern drugs for cancer treatment. Keeping in mind this, a detailed structure-activity relationship study was performed on maslinic acid, a member of the group of triterpenes (oleananes). It is derived from dry olive-pomace oil (an olive skin wax) which is a byproduct of olive oil extraction^5^. It is one of the important anticancer compounds, for which, so far, no three-dimensional quantitative structure–activity relationship (3D-QSAR) study has been yet reported, so that to highlight the key structural controlling regions and different active and inactive sites^6^. Therefore, a 3D-QSAR study was performed on this natural series, to extract out the key regulatory features controlling the anticancer activity and toxicity of maslinic acid. Since no structural information is currently available for maslinic acid in its target-bound state, therefore, a common pharmacophore was developed. This employs molecular field-based similarity method for the conformational search, to design a pharmacophore template which resembles the bioactive conformation. Additionally, the activity-atlas models were generated to get a better insight of structure-activity relationship (SAR). 3D-QSAR also revealed the positive and negative electrostatics regions of the active compounds. This lead, to design more active and optimized analogs of maslinic acid. This 3D-QSAR approach offered a different impact and served as a valuable predictive tool, predominantly in the design of pharmaceuticals^7^. However, getting a good 3D-QSAR is a challenging task. This is, due to the requirements of good and reliable biological data and generation of precise alignments for all compounds with the lowest degree of noise^8^.

Further, predicted compounds were filtered through Lipinski’s rule of five for oral bioavailability evaluation, and ADMET (absorption, distribution, metabolism, excretion, and toxicity) risk assessment. Later screened for synthetic accessibility of predicted compounds. After this, the docking simulation studies were performed on glucocorticoid receptor also known as NR3C1 (nuclear receptor subfamily 3, group C, member 1), a receptor of cortisol and other glucocorticoids^9^. The docking results revealed the putative binding site pocket residues responsible for binding affinity, selectivity, and potency in terms of docking score, comparable to standard inhibitor. Through this study, a primary level understanding of the mode of action of candidate compound P-902 was achieved. Finally, promising compound P-902 was evaluated for detail pharmacokinetics and system pharmacology studies. For the first time, this research article reports the field-based 3D-QSAR modeling, docking, ADMET, and system pharmacology studies on plant derived natural triterpene maslinic acid, leading to identification of major anticancer target, and thereby defining the mechanism of action. Such study would further establish the development of pharmacophore based drug designing, optimization, and drug discovery against breast cancer, a disease affecting millions of lives worldwide.

## Materials and Method

### Parameters for QSAR model development

#### Data collection and Structure preparation

The training dataset of compounds was collected through the prior reports/literature. The two-dimensional (2D) chemical structures were transformed into three-dimensional (3D) structures using the converter module of ChemBio3D Ultra (PerkinElmer/CambridgeSoft, UK).

#### Conformation hunt and Pharmacophore generation

As no structural information is currently available for maslinic acid in its target-bound state, therefore FieldTemplater module of Forge v10 (Cresset Inc., UK) software was used to determine a hypothesis for the 3D conformation. For this, the field and shape information were used by the template through compounds namely, M-159, M-254, M-286, M-543, and M-659. The FieldTemplater-derived hypothesis for the bioactive conformation was then annotated with its calculated field points, resulting in a 3D field point pattern. The field points were generated by using XED (eXtended Electron Distribution) force field. Four different molecular fields such as positive and negative electrostatic, ‘shape’ (van der Waals), and ‘hydrophobic’ (a density function correlated with steric bulk and hydrophobicity) were calculated. The field point pattern provides a condensed representation of the compound’s shape, electrostatics, and hydrophobicity. The XED method was used for the conformational hunt. This employs molecular field-based similarity method for the conformational search to design a pharmacophore template which resembles the bioactive conformation.

#### Compound alignment and 3D QSAR model development

The 3D-QSAR method defines descriptors by calculating the different molecular properties at the intersection points of a 3D frame or grid. This method covers the whole volume of the aligned training set compounds. The pharmacophore template, obtained from the FieldTemplater module was directly transferred into the Forge v10 (Cresset Inc., UK) software, then compounds were aligned with the identified template. Field point based descriptors were used for building 3D-QSAR model after the alignment of 74 compounds with known IC_50_ value onto the identified pharmacophore template. For building the 3D-QSAR model, the maximum number of components were set to 20, the sample point maximum distance was set to 1.0 Å, Y scrambles were set to 50, and also used electrostatic, as well as volume fields. For overall similarity, Forge software uses 50% field similarity and 50% dice volume similarity. The overlays with the best matching low energy conformations, to the template, were taken into consideration for building the 3D-QSAR model. The experimental activity (IC_50_) of the data set compounds were converted to its positive-logarithmic scale by using the formula: pIC_50_ = −log (IC_50_) and defined as the dependent variable. All conformers found were minimized by using the XED force field with a gradient cut-off value of 0.1. The partial least square (PLS) regression method was used through Forge’s field QSAR module^10^. Specifically, the SIMPLS algorithm^11^ was used during QSAR modeling. The initial training set of total 74 compounds was partitioned into a training set (47 compounds) (Table S1) and test-set (27 compounds) (Table S2) to evaluate the QSAR model through activity stratified method.

#### Validation of the QSAR model

The best model was validated by regression coefficient (r^2^), cross-regression coefficient (q^2^), and similarity score (Sim) of conformers for each ligand with respect to the pivot. The derived QSAR model was assessed by leave-one-out (LOO) technique to optimize the activity-prediction model. The LOO cross-validation (LOOCV) is considered one of the most effective methods of regression model validation with small training dataset. Here, training was done with a data size of (N–1) and tested the remaining one, where N represents the complete set of data. In the LOOCV method, the training and testing are repeated for an N amount of time, to pass each data through the testing process^12^. Then derived QSAR model was validated by using test data (not in the training set) (Table S2).

### Visualization of SAR Activity-Atlas models

A Bayesian approach was used to study the global view of training data in a qualitative manner. This approach gives a better understanding of the electrostatics, hydrophobic and shape features, which underlie the SAR of a selected set of compounds. This useful qualitative information was achieved by viewing these models in 3D form. The activity-atlas study revealed the three different types of interrelated biochemical computed data, *i.e*., an average of actives, activity cliffs summary, and regions explored analysis. The average of actives showed the common part in the selected active compounds. The activity cliff summary showed the details about positive and negative electrostatics sites, favorable and unfavorable hydrophobicity, as well as the favorable shape of the active compounds. On the other hand, regions explored analysis showed the regions of the aligned compounds which have been fully explored.

### Prediction set generation and field pattern contribution to the predicted activity

To identify the potential inhibitors, a field point-based virtual screening was performed through the ZINC database^13^. A total of 593 prediction set (query set) compounds were retrieved from the ZINC database, based on Tanimoto score similarity of more than and equal to 80% with that of maslinic acid structure. Furthermore, these compounds were screened through the derived 3D-QSAR model for bioactivity prediction and SAR field point’s compliance. Mismatched SAR field points query/prediction set compounds were removed.

### Lipinski’s Rule of five and Drug like score filtering

Lipinski’s rule of five^14^ was used to screen the prediction set as primary screening step for oral bioavailability. The violating query set predicted active compounds by more than one properties were removed. Later, secondary screening was performed on the basis of drug like score (ADMET risk score and risk parameters) filter through ADMET Predictor^TM^ software (Simulations-Plus Inc., USA). The overall ADMET risk range value set to 0–24 scale. The risk parameters were also provided with a unique quantitative feature, where lower score means, more suitable to a drug like compound^15^. This method was also used to screen out compounds with ADMET risk factor of 10 or more. The ADMET risk parameters were also studied, so that it can be removed or optimized during the experimental chemical synthesis or drug designing process. The identified risk parameters were size, charge, water solubility, volume of distribution, acute rat toxicity, and carcinogenicity, SGOT (serum glutamic oxaloacetic transaminase) elevation, hepatotoxicity, and inhibition of 3 A4 oxidation of midazolam. Standard anticancer drug topotecan was used for comparative study.

### Target identification, docking and synthetic accessibility

#### Target identification

To find out the possible drug targets of the candidate compounds or predicted hits, the STITCH v4.0 (search tool for interactions of chemicals) database, was used as a source to explore known, as well as predicted interactions of chemicals and proteins^16^.

#### Protein preparation

To clean and prepare the target proteins for molecular docking simulations, the 3D protein crystallographic structures, and the coordinates of predicted target proteins were retrieved from the Protein Data Bank (PDB)^17^. Firstly, the protein preparation protocol was used, which perform tasks such as modeling missing loop regions, inserting missing atoms in incomplete residues, deleting alternate conformations and standardizing names of the atoms, protonating titratable residues predicted pKs (a negative logarithmic measure of the acid dissociation constant) and removed the water molecules or hetero atoms. The CHARMM (Chemistry at HARvard Macromolecular Mechanics; Cambridge, MA, USA) force field was used for protein preparation. The hydrogen atoms were added before the processing^18^.

#### Protein-ligand Docking Studies

*In silico* molecular docking simulations and post-docking visualization, studies were performed through the Discovery Studio v3. 5 (Accelrys, USA, 2013), software for molecular modeling^19^. The docking exercise was performed by LibDock program of Discovery Studio molecular modeling software (Accelrys Inc., USA) so that to create the bioactive binding poses of potential inhibitors within the active site of the predicted drug targets. This program uses protein site features referred to as hot spots, and are of two types (polar & apolar). Then this ligand poses were placed into this polar and apolar receptor interactions site. For energy minimization, the Merck Molecular Force Field (MMFF) force field was used in the parameterization step^20^. To generate the conformations, a Conformer Algorithm based on Energy Screening and Recursive build up (CAESAR) method was used. The other docking and scoring parameters kept at their default sets. Further, to identify specific interacting residues of the receptor/target with a bound ligand, a 2D diagram of docking stage was also analyzed. Also, analyzed the protein-ligand complexes, to better understand the interactions between protein residues and bound ligands atoms, along with the binding site residues of the defined or known receptor. The 2D diagrams helped to identify the binding site residues, including amino acid residues, or waters, and/or metal atoms (excluded in this study)^21^. The score ligand poses protocol was used for the scoring functions, such as Jain scoring, LigScore1, the potential of mean force (PMF) and piecewise linear potential (PLP), to evaluate ligand binding ability in a receptor cavity.

#### Synthetic accessibility assessment

To further validate and screened best hit compounds, the synthetic accessibility of the predicted active compounds was measured by using the SYLVIA-XT 1.4 program (Molecular Networks, Erlangen, Germany). It provides a score on a scale from 1 (very easy to synthesize) to 10 (complex and challenging to synthesize). Many criteria, such as the complexity of the molecular structure, number of stereocenters, complexity of the ring system, like commercially available compounds, and the potential for using important synthetic reactions have been independently weighted to provide a single value for synthetic accessibility^22^.

### *In silico* Pharmacokinetics (PK)/Pharmacodynamics (PD) compliance evaluation

The PK/PD properties were calculated by using different standard descriptors to check the compliance of maslinic acid analogs with that of standard drug. The PK covers the study of how the organism affects the drug, while PD covers the study of how a drug affects an organism. The standard pharmacokinetics parameters namely, absorption, distribution, metabolism, excretion, and toxicity (ADMET) were calculated for *in silico* evaluation, so that to prevent late-stage failure of the active lead. Also, evaluate the solubility, ability to be metabolized by cytochrome-P450 (CYP-P450) and metabolism kinetics of the predicted hits (compounds). Metabolism of predicted lead compounds was evaluated in detail by a plethora of diverse enzyme families, involved in xenobiotic metabolisms, such as CYP450 enzymes, dehydrogenases, flavin-containing monooxygenases, hydrolases, peroxidases, UDP-glucuronosyl-transferases (UGTs), sulfotransferases, and glutathione S-transferases. Besides this, the predicted metabolic products and sites of metabolism for Phase-I and Phase-II metabolisms were also estimated through ADMET Predictor^TM^ and MetaPrint2D-React software^23^. The hepatic clearance data was also calculated for evaluation of excretion parameter. Other parameters such as solubility, permeability, the volume of distribution and BBB (blood-brain barrier) were also evaluated for drug-likeness & pharmacokinetics compliance. Lastly, toxicity parameter was evaluated by calculating different standard properties by using ADMET Predictor^TM^ software, which often takes longer time in the drug development process.

## Results and Discussion

### 3D-QSAR modeling on maslinic acid series for anticancer activity against MCF-7 cell line

#### Bioactive conformation hunt and Pharmacophore generation

**Figure 1:**
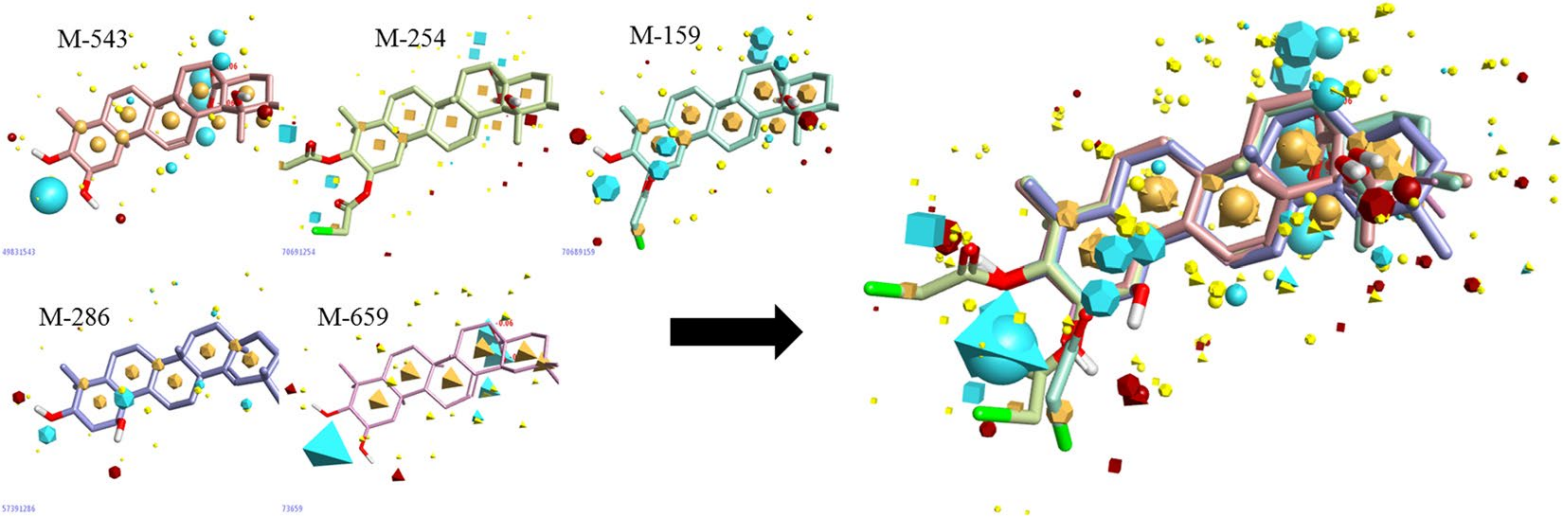
Representation of five template compounds and identification of common pharmacophore bioactive region on the basis of field points for 3D-QSAR model development. Blue color represents negative electrostatic potential, red color represents positive electrostatic potential, orange color represents hydrophobicity and yellow color represents van der Waals descriptors localization.

Prior studies of maslinic acid derivatives showed its promising role in the anticancer activity, but no reports found related to the underlying mechanism of action. Therefore, to shed more lights on this series of compounds, a structure-activity relationship was performed by using molecular field-based 3D QSAR approach. For this, an active conformational hunt was performed on the selected five compounds as a template, namely M-159, M-254, M-286, M-543, and M-659. Due to the lack of 3D protein crystallographic structural data of target protein in complex with maslinic acid, a common pharmacophore was derived (Fig. 1).

**Figure 2:**
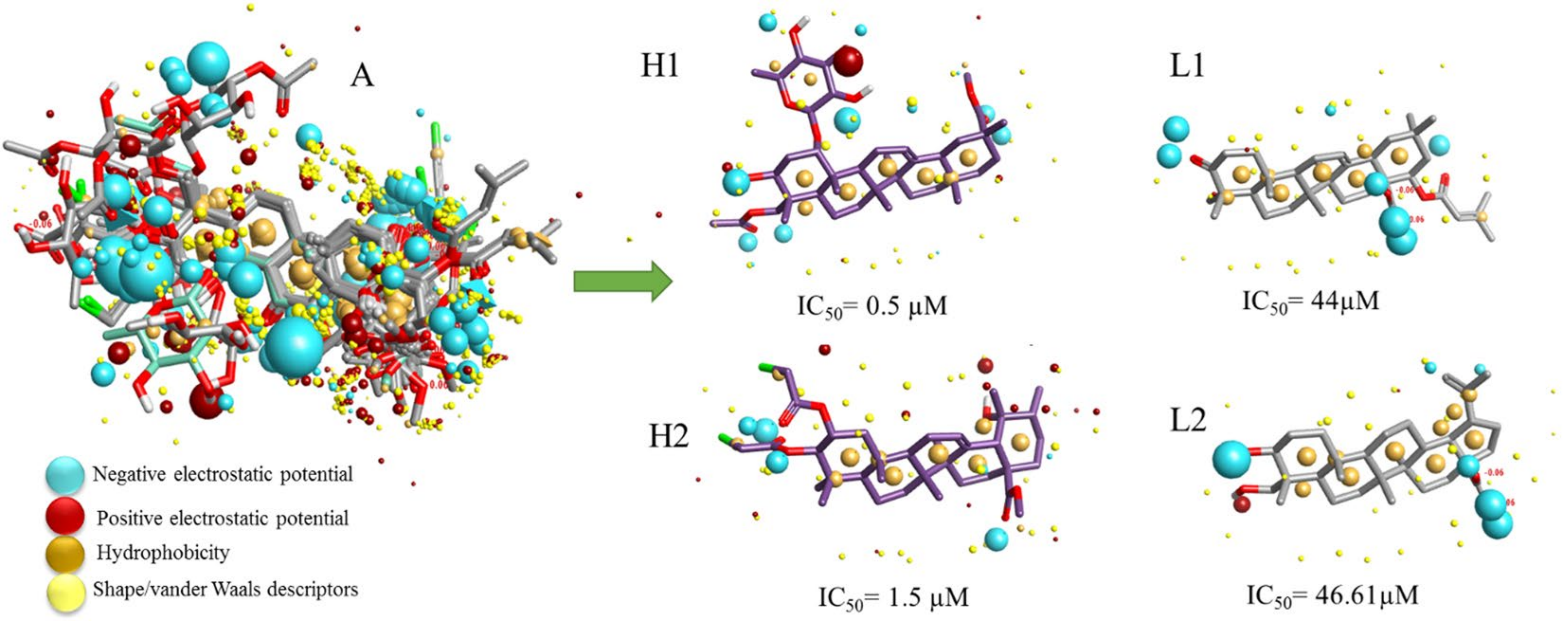
(**A**) Molecular representation of aligned training set compounds with their respective field points. (**B**) Molecular representation of two highly active training set compounds (H1 and H2) and two low active training set compounds (L1 and L2) with their respective biological activity (IC_50_) and molecular field points especially the electrostatic potential regions. Blue color shows negative field points, which indicates likely molecular regions interacting with positive or H-bond donors of target protein, red color shows positive field points, which indicates likely molecular regions interacting with negative or H-bond acceptors of target protein, gold or orange color shows hydrophobic field points, which indicates the regions with high polarizability or hydrophobicity, and yellow color shows van der Waal field points, which indicates the regions with possible surface.

The derived perception of bioactive conformation was then annotated with its calculated field points, resulting in identification of a three-dimensional field points pattern. The field points were generated by using XED force field. Four different molecular fields, namely positive and negative electrostatic potential, shape/van der Waals descriptors, and hydrophobicity (a density function correlated with steric bulk and hydrophobicity) were calculated. This employs molecular field-based similarity method for the conformational search, to design a pharmacophore template which resembles the bioactive conformation, for further virtual screening (Fig. 2).

#### Training set compound alignment and 3D-QSAR model development

**Figure 3:**
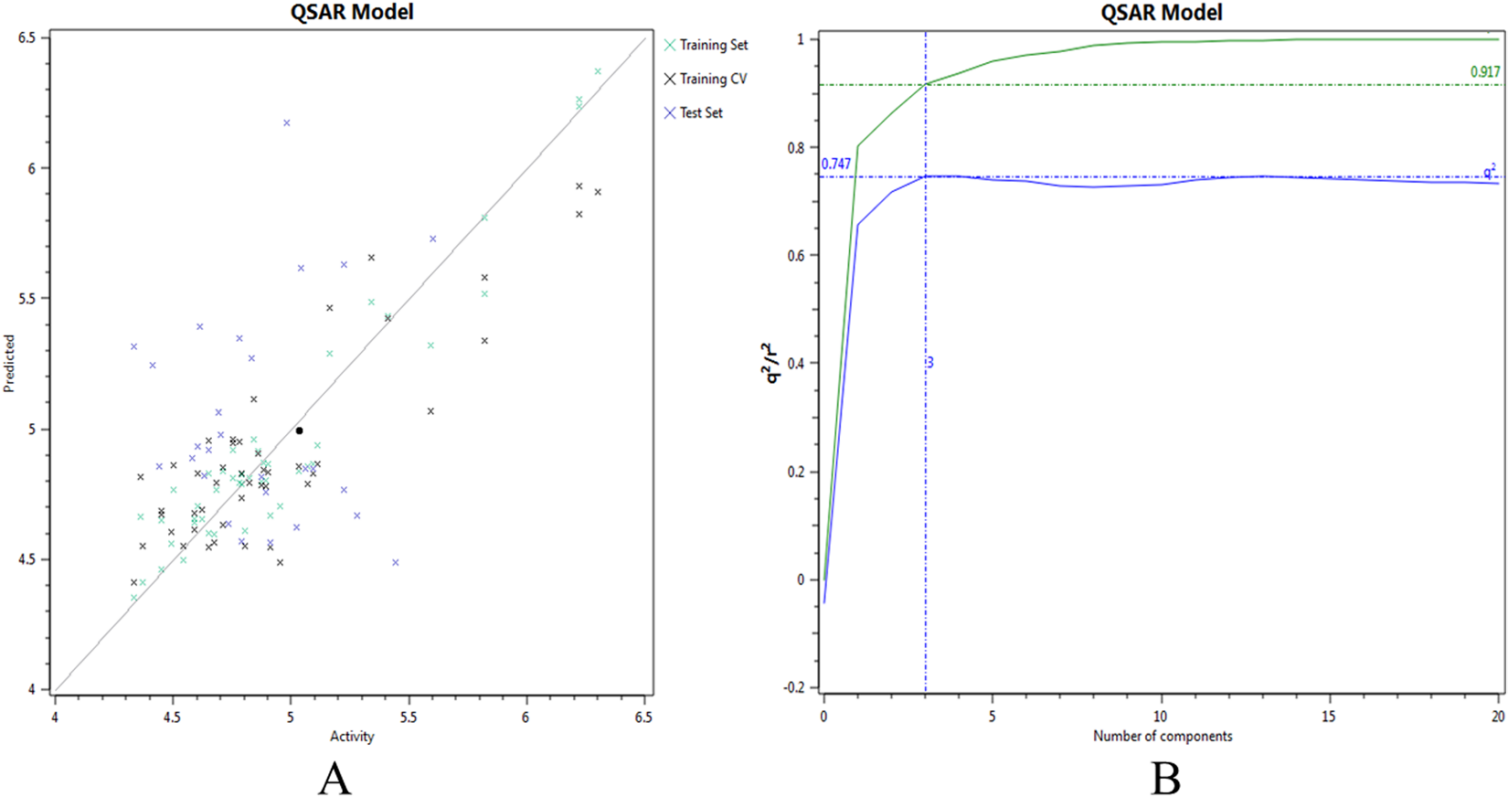
(**A**) Activity interactive graph plot between predicted and actual experimental activity. The graph plot shows separate data series for the training set (green color cross), test set (blue color cross), and training cross-validation set (black color cross). (**B**) 3D-QSAR model performance graph plot between cross-validation regression coefficient, q^2^ (blue color line), and regression coefficient, r^2^ (green color line) ratio and the number of components.

Training set compounds were aligned to the selected pharmacophore template. Field points based descriptors were used to build the 3D-QSAR model after the alignment of 74 compounds. The experimental activity (IC_50_) of the training data set was converted to its positive logarithmic scale by using the formula: pIC_50_ = −log (IC_50_), and defined as the dependent variable. For 3D-QSAR studies, a random method was used for dividing the initial data set into two subsets, *i.e*., 47 compounds in the training set (Table S1), and 27 compounds in the test set (Table S2). Robustness of derived 3D-QSAR model was represented by the activity interactive graph analysis, which shows the predicted versus actual or experimental activity comparison plot with cross-validation data point (Fig. 3A). Moreover, the derived 3D-QSAR model achieved the high activity–descriptors relationship accuracy of 92% as referred by regression coefficient (r^2^ = 0.92) and a high activity-prediction accuracy of 75% as referred by cross-validation regression coefficient (q^2^ = 0.75) (Fig. 3B). Keeping in mind the high descriptive and predictive ability, the derived 3D-QSAR model was considered highly robust prediction or virtual high-throughput screening (vHTS) application tool for the prediction of anticancer/cytotoxic activity of maslinic acid derivatives/analogs against MCF7 breast cancer cell line.

### SAR mechanism of maslinic acid regulated by field points

#### Identification of field points (coefficient & variance) controlling anticancer activity

**Figure 4:**
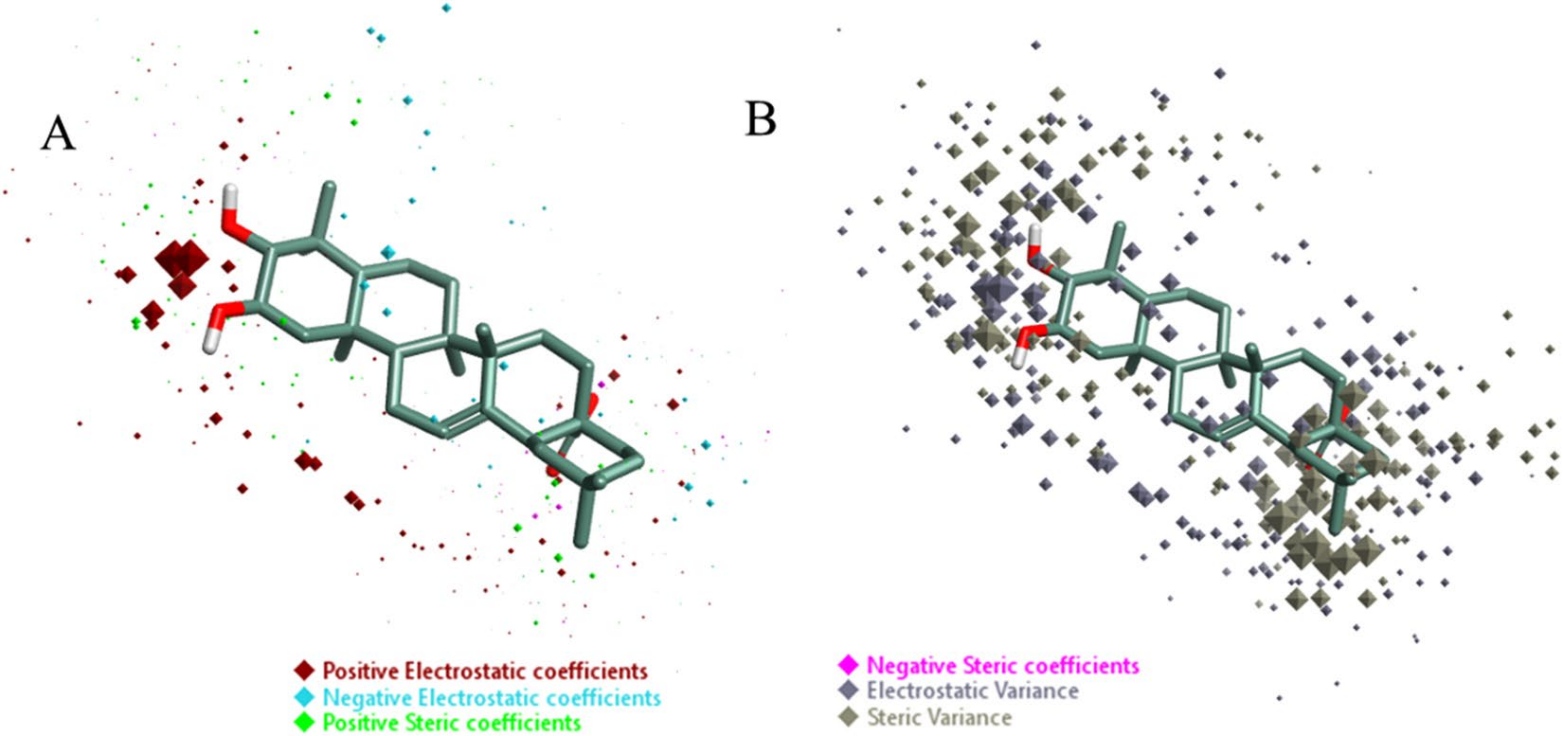
Molecular insight of maslinic acid structure representing the coefficients and variance field points modulating the bioactivity through the derived 3D-QSAR model. (**A**) Model coefficient field points in red color show the region of a strong effect on higher activity. (**B**) High electrostatic variance and high steric variance field points represent the region of high changes and points with low variance indicates the fields in that region with less or no changes.

To unravel the underlying structure-activity relationship (SAR) mechanism of maslinic acid, the derived QSAR model was further visualized in 3D form. For this, training set compound’s bioactivity associated field points, namely coefficient and variance were analyzed in 3D structural form. To better understand the space field points localization, derived QSAR model points were compared with that of reference compound maslinic acid. A high coefficient and a high variance field points were considered truly important correlating parameters in a robust model. Results of structural analysis revealed that the derived QSAR model was well dominated by the electrostatic effects of substituents, as indicated by the large size of red and cyan color and therefore, concluded that electrostatic effects seem to play a minor role in modulating activity, as shown by small size of green and pink field points (Fig. 4).

#### Field contributions to the predicted activity

**Figure 5:**
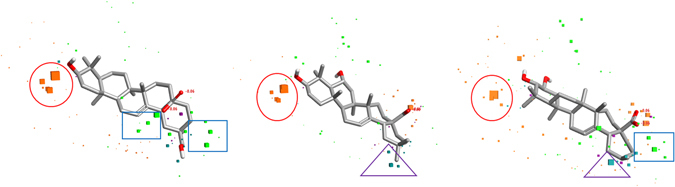
Molecular SAR mechanism of maslinic acid derivatives, representing different geometries of field contributions to the predicted activity. The red color circle indicates electrostatics field points, controlling the decrease in predicted activity. The purple color triangle indicates steric field points, controlling the increase in predicted activity, while blue color square indicates electrostatics field points, controlling the increase in predicted activity.

To evaluate how well maslinic acid and its analogs fits the derived field-based 3D-QSAR model and structural field points regions regulating the predicted activity, ‘view field contributions to predicted activity’ study was performed on maslinic acid and its analogs. These field contributions to predicted activity were shown by a circle (red color), triangle (purple color) and square (blue color) geometries. Results showed that structural area of maslinic acid under the circle, indicates electrostatics field points region with negative regulation ability on predicted activity, *i.e*., to decrease the predicted anticancer activity. On the other hand, the area under the triangle and square, indicate steric and electrostatics field points regions respectively, with positive regulation ability on predicted activity, *i.e*., to increase the anticancer activity (Fig. 5).

### SAR mechanism identification through Activity-Atlas visualization

**Figure 6:**
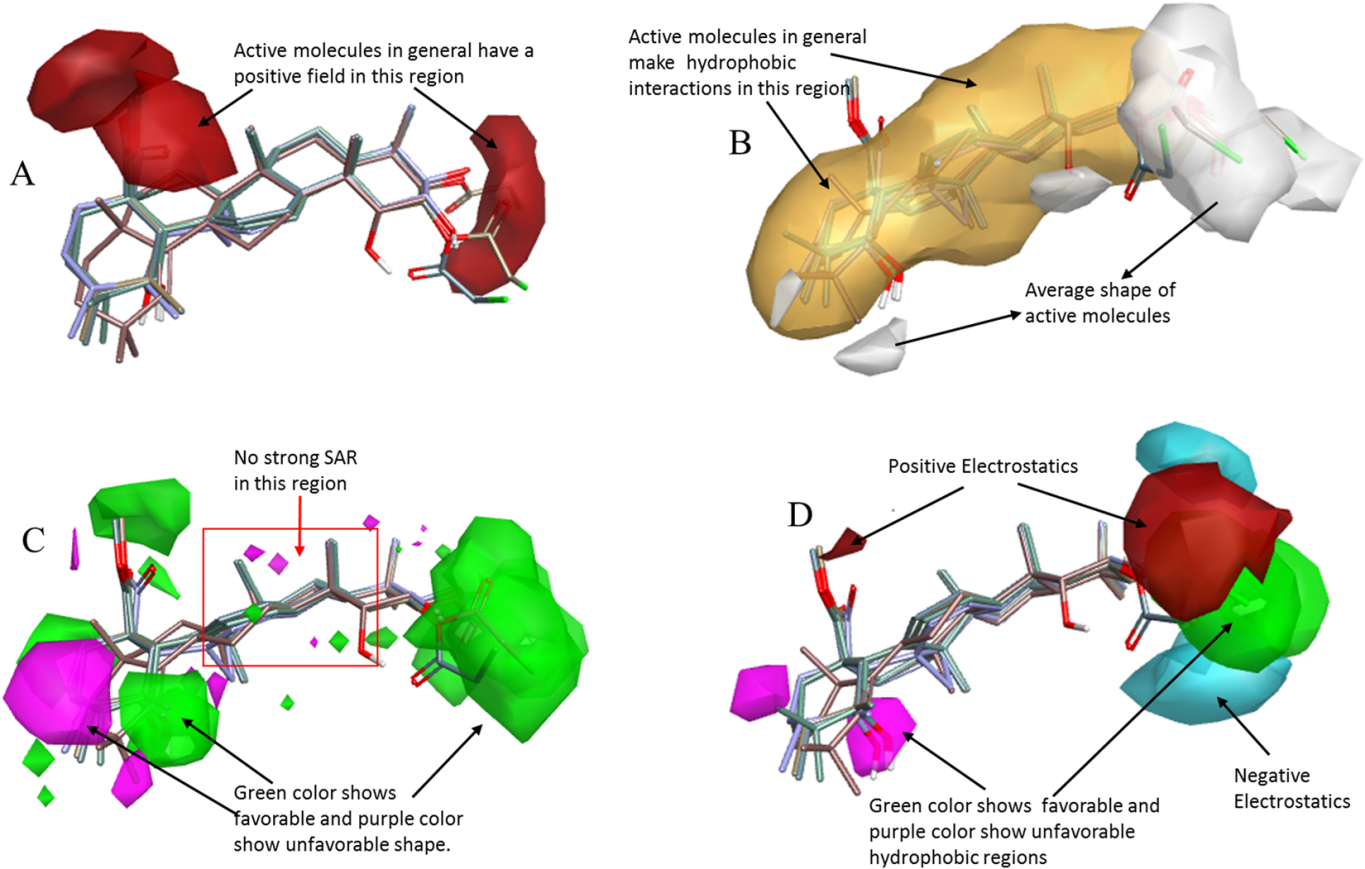
Molecular insight of SAR mechanism models, revealing the different lead optimization sites of active compounds including maslinic acid, as detected through an average of actives and activity cliffs summary studies. (**A**) Red color shows positive field region controlling the activity. High red color region means, higher the activity. (**B**) White color region shows the average shape of active compounds and yellow color shows hydrophobic interactions sites required for activity. (**C**) Green color shows favorable region, purple color shows unfavorable shape region, and red square shows no strong SAR region. (**D**) Red color indicates positive electrostatics region and cyan color indicates negative electrostatics region. Higher the red and cyan color region means higher the activity. Green color shows favorable and purple color shows unfavorable hydrophobic regions controlling the activity.

To reveal the key features of maslinic acid, modulating the anticancer activity for further lead optimization and designing of novel analogs for drug discovery, SAR study was performed through activity-atlas visualization approach. To achieve this, studies related to the average of actives and activity cliffs summary were studied for maslinic acid. Results showed that positive field regions within active SAR model of maslinic acid as indicated by red color sites is regulating the anticancer activity (Fig. 6A). Higher positive field regions (red color site) means higher the cytotoxic/anticancer activity. Modeling results also showed that region of average shape, as indicated by white color region, and hydrophobic interactions regions, as indicated by yellow color are the likely conserved regions controlling the anticancer activity (Fig. 6B). Results also showed that there are favorable (green color) and unfavorable (purple color) shape regions regulating the anticancer activity of active compounds under study. However, 3D-QSAR modeling results also revealed the region with no strong SAR ability (Fig. 6C). Moreover, modeling results also revealed the molecularly conserved sites with positive (red color), as well as negative (cyan color) electrostatics regions positively correlating the anticancer activity, i.e., higher the red and cyan color regions, indicates higher anticancer activity. Beside this, results also revealed the favorable (green color) and unfavorable (purple color) hydrophobic regions controlling the anticancer activity (Fig. 6D).

### Models validation through activity prediction of training and test set

Based on derived SAR models, molecular features controlling the anticancer activity of the active compounds were extracted for further anticancer activity prediction of selected query or prediction data set compounds. Prior to that, prediction performance was firstly evaluated on the training and the test set compounds by predicting anticancer activity through derived models and then compared the distance value (error). For comparison, distinct predicted activity and distance to model columns were evaluated for each derived model. Through this study, essential ligand fields were elucidated for target binding and later these features were used for virtual screening.

### Ligand-based virtual screening for hits prediction

To proposes the hit, a series of field-based 3D similarity-based virtual screening experiments were performed. A set of 593 compounds were identified by using the descriptors of QSAR model. Only high hits compounds with a value of ‘excellent’ were selected. This suggests that most of the features in the compound were found similar to the training set and therefore predicted activities were expected reliable. In contrary, compounds with ‘poor’ field points similarities were removed to avoid consideration of unreliable predicted activities of false positive compounds. Further, high hit compounds were predicted for anticancer activity by using the derived QSAR model. The QSAR model quantified the activity-dependent chemical descriptors and predicted the logarithm of 50% inhibitory concentration (log IC_50_) of each compound, therefore indicated the potential range of inhibition. The compounds with predicted IC_50_ value of more than 20 µM were removed. The potential predicted active compounds were further screened through Lipinski’s rule of five of oral bioavailability (with one rule violation) and later virtually screened through ADME parameters and toxicity risk of drug-likeness (Table S3).

### Identification of ADMET risk range and risk parameters

The ADMET risk score was calculated to identify the real ADMET problems behind the predicted lead compounds so that to prevent the failure of the compound during clinical studies. This method was also used to screen out compounds with ADMET risk factor of 10 or more. The lower ADMET risk score is a mostly preferable step in drug discovery process. The parameters of the risk were also studied so that it can be removed during designing of drugs. The predicted compound P-902 showed a risk of 4.22 in compare to control anticancer drug topotecan, which showed a score of 2.0 (Table 1). The risk parameters of predicted hit compound under study were due to its size, charge, water solubility, the volume of distribution, acute rat toxicity, and carcinogenicity, SGOT elevation, hepatotoxicity, and inhibition of 3A4 oxidation of midazolam (Table S3). Results showed that predicted active compound P-902 may require more lead optimization to fits well with standard drug range.

**Table 1:** Details of predicted *in silico* ADMET risk range and risk parameters of compound P-902 and standard anticancer drug topotecan.

Risk	Absorption	P450 oxidation	Mutagenicity	Toxicity	ADMET Risk	Risk Parameters
Range	**0–8**	**0–6**	**0–4**	**0–7**	**0–24**	
P-902	3.26	0.0	0.0	0.96	4.22	Size, charge, water solubility, lipophilicity
Topotecan (control)	0.0	0.0	2.0	2.0	2.0	Hepatotoxicity, inhibition of 3A4 oxidation of midazolam

### Assessment of predicted hits for synthetic accessibility

To ensure the viable virtual screening, predicted compounds were evaluated for synthetic accessibility. Later, results of synthetic accessibility of predicted leads were compared with that of standard anticancer drug doxorubicin. Results showed synthetic accessibility of predicted hits, similar to standard drug doxorubicin. The SYLVIA scores of hits and standard drug were found similar and therefore results of hits indicate the synthetic ability of predicted leads.

### Potential anticancer drug targets identification

Following the development of the QSAR model, hits prediction, filtering through the Lipinski’s rule of five for oral bioavailability and ADME compliance evaluation, toxicity risk assessment and synthetic viability, best hits or predicted active maslinic acid analogs were further evaluated for target binding affinity through molecular docking simulation studies. Prior to docking, potential cellular targets of predicted maslinic acid analogs (best hits) were identified through the STITCH v4.0 software. Through this, potential anticancer targets were predicted, such as aldo-ketoreductase family-1 member B10 (AKR1B10)^24^, nuclear receptor subfamily-3 group-C member-1 (NR3C1)^9^, and prostaglandin-endoperoxide synthase (PTGS2)^25^ (Figure S1). Besides, predicted best hits were also evaluated by docking against known anticancer target, *viz*., human epidermal growth factor receptor 2 (HER2), mostly expressed in about 25% cases of breast cancer patients^26^. The primary purpose of docking simulation study is to elucidate whether the identified best hit compounds able to bind and regulate the anticancer targets/receptors and also to study their possible mechanism of action and measure the binding affinity. For this, predicted best hits were evaluated by docking studies against identified anticancer targets. None of the predicted compounds docked with HER2 (PDB ID: 3PP0) and PTGS2 (PDB ID: 4FM5) but showed low docking score with AKR1B10 (PDB ID: 1ZUA). However, predicted best hit maslinic acid analogs well docked with NR3C1 (PDB ID: 4UDD), a known anticancer target and reported to have a role in promoting cancer cell survival and induce chemoresistance in breast cancer patients^9^. The 3D crystallographic protein structure of NR3C1 with a resolution of 1.8 Å was retrieved from PDB database. The NR3C1 also known as glucocorticoid receptor (GR or GCR) is a receptor for binding of cortisol and other glucocorticoids. The NR3C1 can function both as a transcription factor that binds to glucocorticoid response elements in the promoters of glucocorticoid responsive genes to activate their transcription, and as a regulator of other transcription factors. This is found in cytoplasm, but transported to nucleus after ligand binding. The NR3C1 involved in inflammatory responses, cellular proliferation, and differentiation in target tissues^27^. Prior studies suggest role of NR3C1 against cancer such as, acute lymphocytic leukaemia (ALL), prostate cancer, multiple myeloma, and stomach cancer (http://www.cancerindex.org)^28^. Results of molecular docking with target NR3C1 revealed that except predicted compounds, *viz*., P-902 and P-701, rest showed low binding affinity docking score in compared to control co-crystallized inhibitor CV7, therefore both the predicted compounds were considered best-predicted leads.

**Figure 7:**
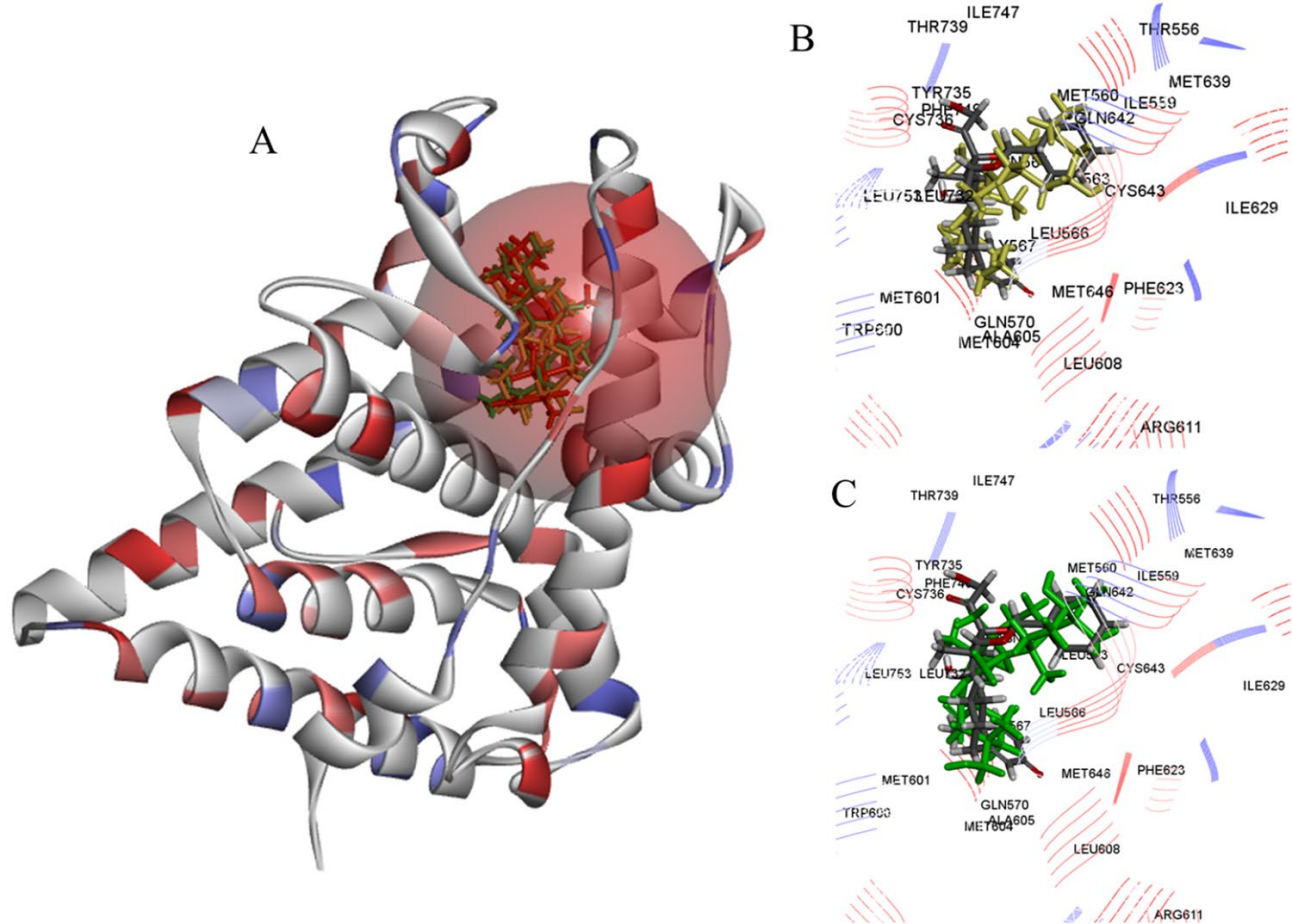
(**A**) Protein crystallographic structural model of human NR3C1 (PDB ID: 1ZXM) with the co-crystallized inhibitor CV7 binding site (orange color sphere). (**B**) Binding site pocket residues with best fit confirmation of maslinic acid analog P-902 (yellow color). (**C**) Binding site pocket residues with docked maslinic acid analog P-902 (green color) and control drug CV7.

Potential of these best hit compounds was measured through LibDock docking score. The best hit compounds (maslinic acid analogs), namely, P-902 & P-701, when docked with NR3C1 showed the LibDock scores of 123.691 & 103.797, respectively, which were found lower but under the limit to control drug CV7 docking (LibDock) score of 167.349. Molecular docking results of predicted active compound P-902 revealed two hydrogens (H) bonds with their binding site amino acid residue Tyr_735_ and single H-bond with amino acid residue Met_601_. On the other hand, no H-bond found with the binding site residues for predicted best hit compound P-701. However, control drug CV-7 showed three H-bonds formation with the binding site amino acids, namely Gln_570_, Thr_739_, and Asn_564_. In the studied work, optimum orientations (*i.e*., most active confirmations) of candidate compounds were also evaluated (Fig. 7). The inhibitory activity of P-902 was explained by two major factors: docking score and H-bond interactions. The docking reliability was validated by using the experimentally known crystallized X-ray 3D structure of target protein receptor complex from PDB database. For docking protocol optimization and validation confidence, the co-crystallized drug (i.e., CV-7) was re-docked on to the known binding site and the docked conformations with the highest docking score were selected as the most probable active binding confirmation. Results showed that docked compounds were almost in the same position with that of co-crystallized drug CV-7. Further, docking experiments for binding affinity evaluation of predicted hits were measured in terms of different standard scoring functions, such as LigScore1, PLP2, Jain score, PMF, and PMF04, and then compared with that of positive control drug (Table 2). Besides, molecular insight was revealed by 2D diagram analysis, which showed various molecular interactions, such as H-bonds, atomic charge interactions, and Pi-sigma interactions between the ligand and the surrounding residues, so that to unravel the molecular mechanism of action behind activity & stable binding specificity. The presence of covalent bond means higher chances of therapeutic activity and binding stability, and mostly these compounds refer as ‘covalent inhibitors’. In 2D diagram results, for more clarity, different colors were used to highlight the different molecular interactions, *e.g*., pink color indicates electrostatic interaction, purple color indicates covalent bond, and green color means van der–Waals molecular interaction. Beside this, solvent accessibility of the ligand atom and the amino acid residues was represented by light blue color shades surrounding the respective atoms or residues. High shades indicate more exposure to solvent (Figure S2).

**Table 2:** Details of LibDock scoring functions, H-bond and interacted binding site amino acid residues for maslinic acid analogs & control drug docked on anticancer target NR3C1.

Compound ID	LibDock Score	H-bond	LigScore1	PLP2	Jain score	PMF	PMF04	Amino acid residues
P-902	123.691	Tyr_735_(2) Met_601_	6.7	101.12	10.88	157.86	22.66	Thr_556_, Ile_559_, Met_560_, Leu_563_, Asn_564_, Gly_567_, Gln_570_, Trp_600_, Met_601_, Met_604_, Ala_605_, Leu_608_, Phe_623_, Ile_629_, Met_639_, Gln_642_, Cys_643_, Met_646_, Leu_732_, Tyr_735_, Cys_736_, Phe_749_, Leu_753_
P-701	103.797	No	5.11	92.47	8.09	145.41	16.61	Thr_556_, Ile_559_, Met_560_, Leu_563_, Asn_564_, Gly_567_, Gln_570_, Trp_600_, Met_601_, Met_604_, Ala_605_, Leu_608_, Leu_621_, Phe_623_, Ile_629_, Met_639_, Gln_642_, Cys_643_, Met_646_, Leu_732_, Tyr_735_, Cys_736_, Leu_753_
CV7 (control, inhibitor)	167.349	Gln_570_ Thr_739_ Asn_564_	7.85	139.78	12.63	174.38	45.28	Thr_556_, Ile_559_, Met_560_, Leu_563_, Asn_564_, Leu_566_, Gly_567_, Gln_570_, Trp_600_, Met_601_, Met_604_, Ala_605_, Leu_608_, Arg_611_, Phe_623_, Ile_629_, Met_639_, Gln_642_, Cys_643_, Met_646_, Leu_732_, Tyr_735_, Cys_736_, Thr_739_, Ile_747_, Phe_749_, Leu_753_

### *In silico* PK/PD analysis for compound P-902

The most potent maslinic acid analog and best-hit compound P-902 was further evaluated for *in silico* pharmacokinetics parameters (*i.e*., absorption, distribution, metabolism, excretion, toxicity; ADMET) compliance with standard range. The calculated pharmacokinetics parameters of maslinic acid analog P-902 were compared with that of standard anticancer drug topotecan (Table S4). The calculated results of ADMET studies showed that compound P-902 was slightly lipophilic in nature with good solubility but slightly lower than topotecan. The calculated molecular diffusion coefficient (in water) for best hit maslinic acid analog P-902 was 0.539 cm^2^/s × 10^5^ and octanol-water distribution coefficient (LogP/D) was 5.858. The compound P-902 showed a tendency to supersaturate in water and revealed native water solubility of 0.008 mg/mL. Likewise, the calculated solubility of compound P-902 in a fasted state at gastric fluid was 4.015E-4 mg/mL, and in a fasted state at intestinal fluid was 0.079 mg/mL, whereas in a fed state at intestinal fluid it was 0.201 mg/mL. The compound P-902 was also analyzed for permeability compliance, a key determinant factor in ADMET studies or prior to clinical trials, with the help of human skin and human jejunal effective permeability parameters, along with apparent Madin-Darby Canine Kidney Cells-On-Sheet (MDCK COS) permeability, and permeability through rabbit cornea. The permeability through human skin was 113.623 cm/s × 10^7^ and the Peff (effective jejunal permeability) was found to be 3.686 cm/s × 10^4^. The MDCK permeability was 50.684 cm/s × 10^7^, and the permeability through rabbit cornea was 224.475 cm/s × 10^7^. Moreover, ADMET results of predicted best-hit compound P-902 revealed liver high intrinsic passive uptake capacity, which is considered safe in sense of pharmacology studies. Also, calculated the volume of distribution which was detected 0.477 L/kg. The compound P-902 showed low ability to cross the BBB. The brain/blood partition coefficient was detected (in logarithm) −0.649, whereas the percent unbound to blood plasma proteins was detected 1.657. The blood to plasma concentration ratio was predicted to be 0.57 for compound P-902.

The major cytochrome family enzymes involved in the metabolism of compound P-902 were also predicted by using structural data. Results revealed that compound P-902 was a substrate of CYP1A2, CYP-2C8 and CYP3A4 in human and indicate general inhibitory action against CYP2C9 and CYP3A4. The specifically predicted sites of human CYP2C8 mediated oxidation were C33(897), C20(869), C1(642), while the predicted sites of human CYP3A4 mediated oxidation were C33(768), C3(678), C20(551), C1(545) and C18(478). The compound P-902 was found to be the inhibitor of the CYP3A4-mediated metabolism of midazolam and the predicted inhibition constant (Ki) value for midazolam inhibition was 46.028 µM. In identifying the affinity of compound P-902 with CYP-P450 enzymes in quantitative terms, the Km value and Vmax were calculated, which provide the knowledge of the rate of metabolism. The kinetic Michaelis-Menten Km constant for predicted sites of CYP3A4 mediated metabolism was 51.402 µM, whereas the Vmax constant for predicted sites of enzyme CYP3A4 mediated metabolism was 6.456 nM/min/nM. The intrinsic clearance constant (Clint) for predicted sites of CYP3A4 mediated metabolism was 13.940 µL/min/mg. The kinetic Michaelis-Menten Km constant value for CYP3A4 mediated metabolism from human liver microsomes was 51.168 µM, while Vmax constant for predicted sites of CYP3A4 mediated metabolism from human liver microsomes was 2.344 nM/min/nM. The intrinsic clearance constant for predicted sites of CYP3A4 mediated metabolism of human liver microsomes was 45.813 µL/min/mg. The derived metabolism kinetics data may be used to calculate the hepatic clearance and also for the *in vitro* and *in vivo* relationship. The overall intrinsic clearance in human liver microsomes was, 11.051 μL/min/mg for compound P-902, a maslinic acid analog. Through the metabolic rate information of other metabolites, precise knowledge of elimination rate can be used to calculate the candidate drug’s half-life and total clearance (Table S5).

**Figure 8:**
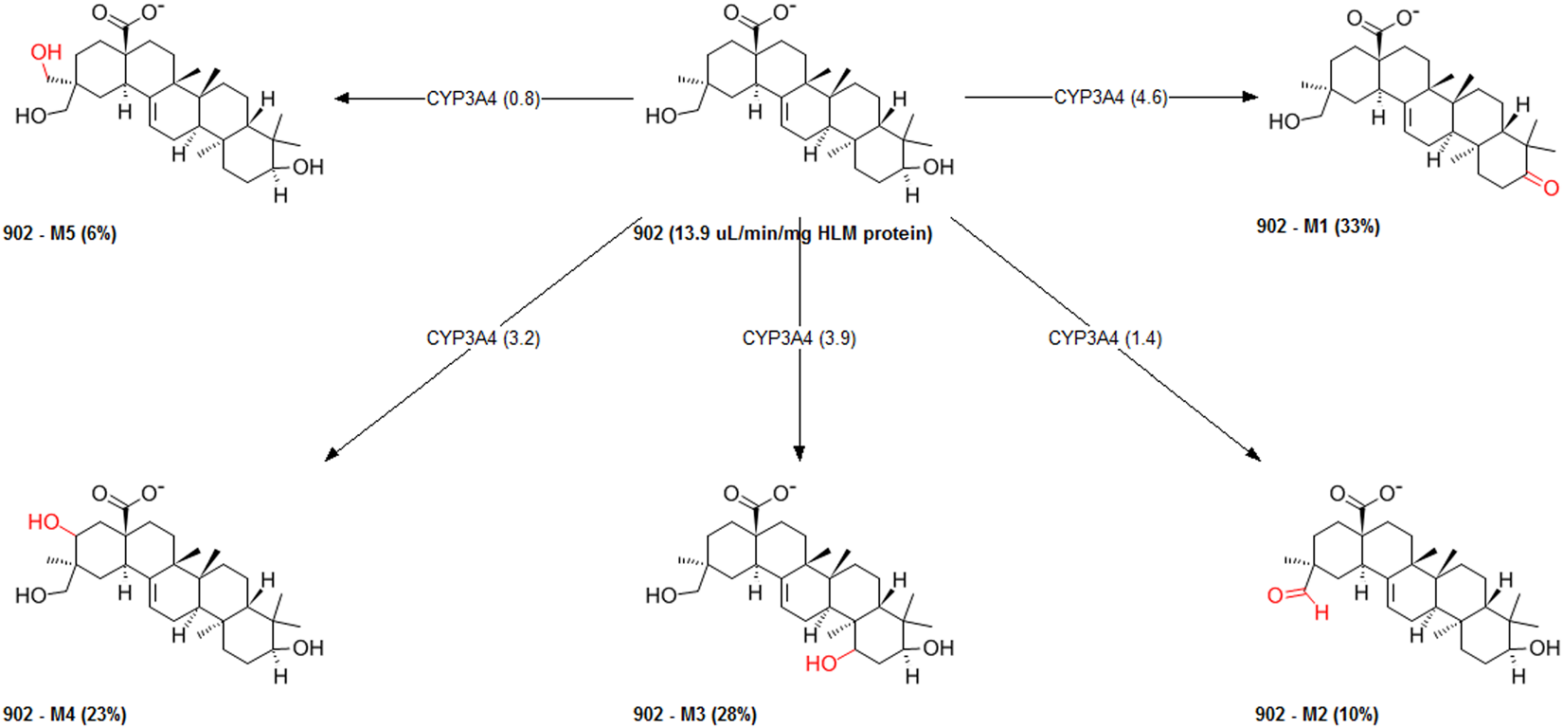
The possible metabolites of candidate compound P-902.

**Figure 9:**
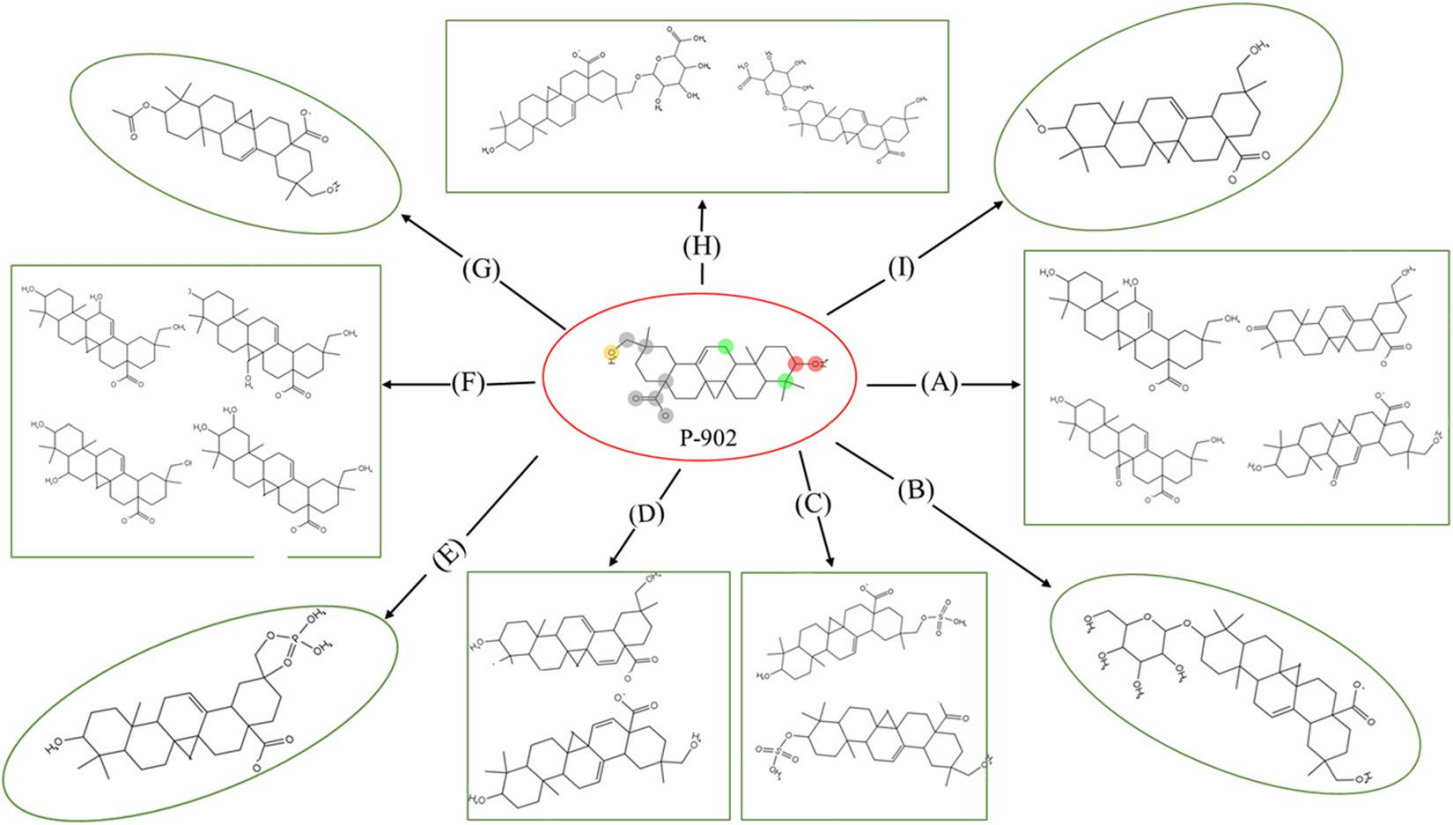
The possible phase II metabolites of candidate compound P-902 developed through different biochemical reactions. (A) Oxidation, (B) Glucosidation, (C) Sulfation, (D) Aromatization, (E) Phosphorylation, (F) Hydroxylation, (G) Acetylation, (H) Glucuronidation, and (I) Methylation.

In addition to the cytochrome P450s, there is variety of other drug metabolizing enzymes that can affect how much of an orally administered drug reaches the systemic circulation including other oxidases, hydrolases, reductases, and dehydrogenases (oxidoreductases). The drug metabolism is commonly divided into two phases; phase-I (or functionalization reactions) and phase-II (conjugative reactions). The reactions of phase-I are thought to act as a preparation of the drug for the phase-II reactions. This drug metabolism produces several metabolites which may have different pharmacological and physicochemical properties. The study of predicting potential metabolism-mediated drug interactions of derived metabolites may show significant implications for both drug efficacy and safety. In this perspective, computational approaches were used for investing drug metabolism in identifying the effects of compound P-902 and its biotransformation pathway for phase-I functionalization reactions and phase-II biosynthetic reactions. To study this, metabolic site, metabolites, and type of reactions involved in candidate compound P-902 were studied in detail. Details of cytochrome P450 mediated metabolites of compound P-902 were summarized (Fig. 8). Additionally, possible metabolic sites and metabolites were predicted for different biochemical reactions through oxidation, glucosidation, sulfation, aromatization, phosphorylation, hydroxylation, acetylation, glucuronidation and methylation (Fig. 9).

Moreover, compound P-902 also showed glucuronidation potential by uridine 5′-diphospho-glucuronosyltransferases 1A3 and 2B7 enzymes, which have the ability to transform the small molecules to water soluble form. Since the compound P-902 was slightly lipophilic in nature, thus its renal clearance will decrease, while metabolic clearance may increase. These metabolites may be helpful in designing new compounds, as well as to optimize the pharmacological effects.

### *In silico* toxicity risk assessment for compound P-902

The safety of the lead compound is an important parameter for a successful drug. The candidate compounds P-902 predicted to be a non-biodegradable and therefore evaluated for different toxicity parameters and side effects so that to minimize the failure during clinical studies. The maximum recommended therapeutic dose for the compounds P-902 was predicted to be below 3.16 mg/kg/day. The compound P-902 was found non-allergenic, respiratory sensitizer and non-skin sensitizer. Regarding hepatotoxicity, compound P-902 was evaluated through different liver enzymes. Results showed that compound P-902 may elevate some of the liver associated enzymes, such as alkaline phosphatase, but at the same time, no effect observed in the GGT (gamma-glutamyltransferase), SGOT & SGPT (serum glutamate-pyruvate transaminase) level, except LDH (lactate dehydrogenase) enzymes. Results suggest that candidate compound P-902 may not cause the phospholipidosis, developmental or reproductive toxicity, and estrogen receptor toxicity in rats, but may cause the androgen receptor toxicity in rats, either through original form or metabolites. Toxicity results also showed no chance of triggering the mutagenic chromosomal aberrations and showed negative mutagenicity, when evaluated through the TA97, TA1537, and TA98 strains of *Salmonella typhimurium* and the TA100, TA102, TA1535, WP2 uvrA strains of *Escherichia coli*. The calculated carcinogenic potency of compound P-902, measured in terms of tumorigenic dose 50% (TD_50_) for the rat was 84.385 mg/kg/day and for the mouse, it was 381.422 mg/kg/day. The calculated lethal dose 50% (LD_50_) for acute rat toxicity was 204.531 mg/kg. Results suggest that compound P-902 was within the limit of standard toxicity parameters range and suitable for further drug discovery stages. These findings will be helpful to set dose ranges for *in vivo* animal assays, and in planning future chemical synthesis for pharmaceutical product/formulation development. besides this, the negative decimal logarithm of the 50% lethal concentration (pLC_50_) for *Daphnia magna* (water flea), the calculated lethal toxicity after 48 hours of exposure of candidate compound P-902 was 0.893 mg/L, and the 50% lethal concentration (LC_50_) for Fathead minnow, the calculated lethal toxicity after 96 hours of exposure was 0.31 mg/L. However, these predicted toxicities can be minimized by the help of dose limit studies. Moreover, the candidate compound P-902 was predicted to be non-toxic for estrogen receptor, but seems toxic for the androgen receptor and also may not cause phospholipidosis (Table 3).

**Table 3:** Details of calculated toxicity risk parameters for compound P-902 and control drug topotecan.

Properties		P-902	Topotecan
Maximum recommended therapeutic dose, administered orally		Below 3.16 mg/kg/day	Above 3.16 mg/kg/day
Estrogen receptor (rats)		Non-toxic	Toxic
Quantitative measure of estrogen receptor toxicity in rats		Non-toxic	4.3531
Androgen receptor toxicity		Toxic	Toxic
Quantitative measure of androgen receptor toxicity in rats		0.037	0.0599
Allergenic skin sensitization (mice)		No sensitization	No sensitization
Allergenic respiratory sensitization in rat		No sensitization	No sensitization
Fathead minnow lethal toxicity after 96 hours of exposure		0.31 mg/L	1.5 mg/L
*Daphnia magna* (water flea) lethal toxicity after 48 hours of exposure		0.893 mg/L	0.549 mg/L
Bio concentration factor		3.781	4.688
Biodegradation		No	No
Likelihood of the hERG potassium channel inhibition in human		No (95%)	No
Affinity towards hERG K+ channel and potential for cardiac toxicity		5.060 mol/L	5.24 mol/L
LD_50_ for lethal rat acute toxicity		204.531 mg/kg	782.92 mg/kg
Tumorogenic dose, rat		84.385 mg/kg/day	9.94 mg/kg/day
Tumorogenic dose, mice		381.422 mg/kg/day	74.25 mg/kg/day
Triggering the mutagenic chromosomal aberrations		Non-toxic	Toxic
Hepatotoxicity	levels of Alkaline Phosphatase enzyme	Elevated	Elevated
	levels of GGT enzyme	Normal	Normal
	levels of LDH enzyme	Elevated	Normal
	levels of SGOT enzyme	Normal	Elevated
	levels of SGPT enzyme	Normal	Normal
Causing phospholipidosis		Non-toxic	Non-toxic
Reproductive/developmental toxicity		Non-toxic	Non-toxic
Mutagenicity (pure compound)	TA97 and/or TA1537 strains of *S. typhimurium*	Negative	Positive
	TA98 strain of *S. typhimurium*	Negative	Negative
	TA100 strain of *S. typhimurium*	Negative	Negative
	*S. typhimurium* and/or WP2 uvrA strain of *E. coli*	Negative	Positive
	TA1535 strain of *S. typhimurium*	Negative	Negative
Mutagenicity (microsomal rat liver metabolites)	TA97 and/or TA1537 strains of *S. typhimurium*	Negative	Positive
	TA98 strain of *S. typhimurium*	Negative	Negative
	TA100 strain of *S. typhimurium*	Negative	Negative
	TA102 strain of *S. typhimurium*	Negative	Negative
	TA1535 strain of *S. typhimurium*	Negative	Negative

Abbreviations: LD_50_, lethal dose 50%; hERG, human Ether-a-go-go-Related Gene; SGOT, serum glutamic oxaloacetic transaminase; SGPT, serum glutamate-pyruvate transaminase; GGT, gamma-glutamyl transpeptidase; LDH, lactate dehydrogenase.

## Conclusion

The virtually screened compound P-902 was found best fit maslinic acid analog clearing all filters, and predicted to be cytotoxic/anticancer active against human breast cancer cell line MCF7. A field based 3D-QSAR model was proposed as a virtual screening tool for the identification of anticancer active or optimized leads against breast cancer. This 3D-QSAR model defines the molecular level understanding and regions of structure-activity relationship for triterpene maslinic acid and its analogs. The key features such as average shape, hydrophobic regions and electrostatic patterns of active compounds were mined and mapped to virtually screen potential analogs. The field point based descriptors were used for development of 3D-QSAR model by aligning the training set compounds onto identified pharmacophore template. The derived PLS regression QSAR model showed the acceptable regression coefficient of 0.92 and cross-validation coefficient of 0.75. For better understanding of the electrostatics, hydrophobic, and shape features responsible for SAR, a global view of training set was studied by using Bayesian approach and later visualized as the activity-atlas models, which revealed the average of actives, activity cliffs summary, and regions explored. A total of 593 prediction set compounds (with more than 80% structural similarity to maslinic acid) were virtually screened with the help of derived QSAR model, as potential hits retrieved from the ZINC database. After virtual screening through Lipinski’s rule of five filter for oral bioavailability, ADMET risk filter for drug like features, and synthetic accessibility filter for chemical synthesis, out of 593 hits, only 39 top hits were found under limit. Later, AKR1B10, NR3C1, PTGS2, and a known anticancer target HER2 were identified as potential cellular targets for docking. Based on high binding affinity or low energy docking scores, out of 39 hits, only two compounds namely, P-902 and P-701 were found best fit against NR3C1, a glucocorticoid receptor. After comparing all screening parameters, compound P-902 was found top most hit. Results showed that identified compound P-902 was under standard limit of *in silico* PK/PD, and toxicity risk parameters, by comparing with anticancer drug topotecan. These, results may be of great help in early anticancer drug discovery, and lead optimization from natural active scaffold.

## Electronic supplementary material

Supplementary information
