# Supplementary material for: 3D-QSAR studies on Maslinic acid analogs for Anticancer activity against Breast Cancer cell line MCF-7
Source: Sci Rep. 2017 Jul 20;7:6019. doi: 10.1038/s41598-017-06131-0 (PMC5519539; doi:10.1038/s41598-017-06131-0)
Supplement: Supplementary file 1 — Supplementary information [file 41598_2017_6131_MOESM1_ESM.pdf]

3D-QSAR studies on Maslinic acid analogs for Anticancer activity against Breast Cancer cell line MCF-7

Sarfaraz Alam[1, 2] and Feroz Khan[1, 2,*,#]

[1]Metabolic & Structural Biology Dept., CSIR-Central Institute of Medicinal & Aromatic Plants, P.O.-CIMAP, Lucknow-226015 (U.P.), INDIA
[2]Academy of Scientific & Innovative Research (AcSIR), CSIR-CIMAP Campus, Lucknow-226015 (U.P.), INDIA

*Correspondence to be addressed:
Metabolic & Structural Biology Department, CSIR-Central Institute of Medicinal & Aromatic Plants, P.O.-CIMAP, Kukrail Picnic Spot Road, Lucknow-226015 (Uttar Pradesh), India; Tel.: +91 522 271 7668, Fax: +91 522 234 2666; E-mail: f.khan@cimap.res.in; CIMAP Communication No.: CIMAP/PUB/2016/85.

[#]Present Address: *Skaggs School of Pharmacy & Pharmaceutical Sciences, University of California San Diego (UCSD), 9500 Gilman Drive, La Jolla, San Diego, CA 92093, USA*

Table S1. Details of the training set compounds with experimental and predicted anticancer activities.

Serial No.	Compound	Structure	Experimental activity (pIC_{50})*	Predicted Activity (pIC_{50})*	Error factor**
1	T-254	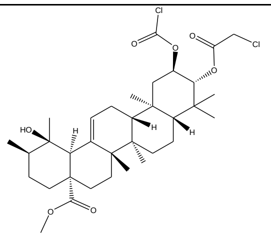	5.82	5.5	-0.32
2	T-971	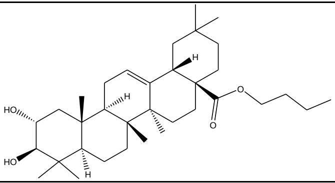	4.88	4.9	0.02
3	T-623	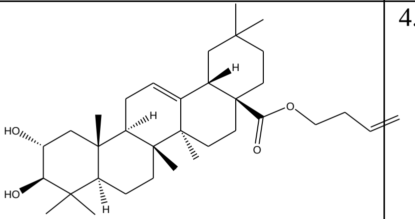	4.9	4.9	0

4	T-159	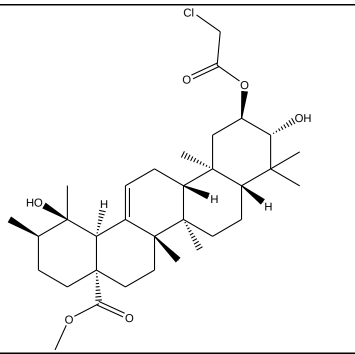	5.59	5.3	-0.29
5	T-418	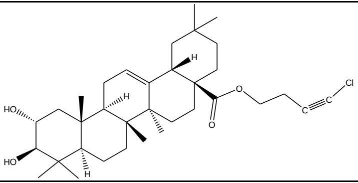	5.11	4.9	-0.21
6	T-013	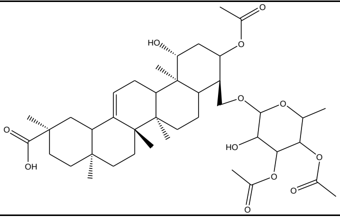	5.82	5.8	-0.02
7	T-689	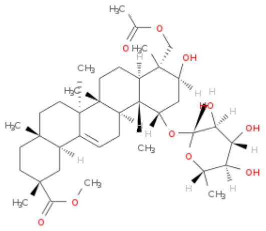	6.3	6.4	0.1
8	T-726	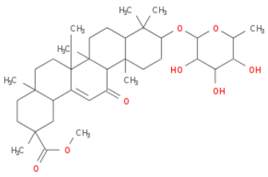	4.71	4.6	-0.11
9	T-598	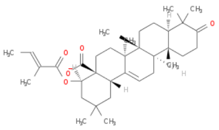	4.59	4.6	0.01

10	T-585	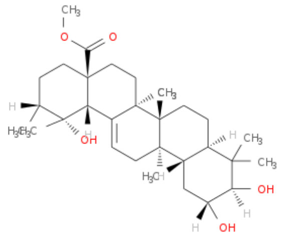	4.5	4.8	0.3
11	T-494	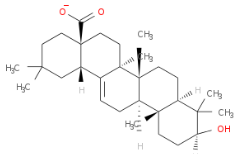	4.45	4.7	0.25
12	T-756	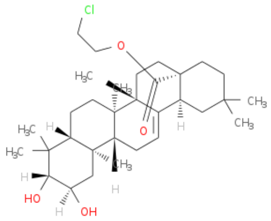	4.75	4.9	0.15
13	T-816	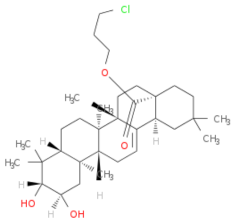	4.86	4.9	0.04
14	T-250	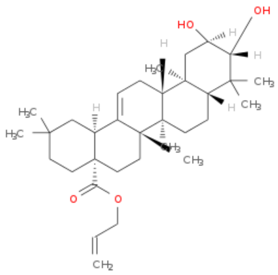	4.82	4.8	-0.02
15	T-066	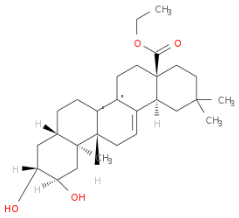	4.84	5	0.16

16	T-863	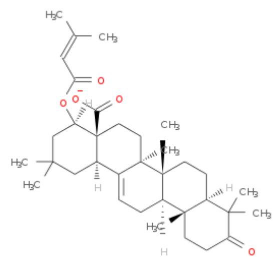	4.36	4.7	0.34
17	T-542	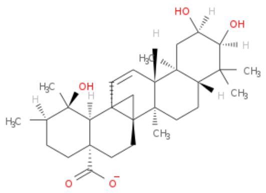	4.8	4.6	-0.2
18	T-676	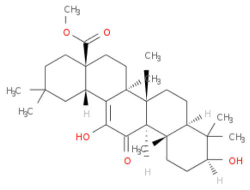	4.59	4.7	0.11
19	T-770	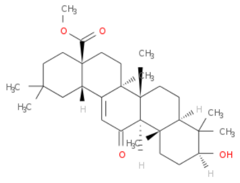	4.67	4.6	-0.07
20	T-361	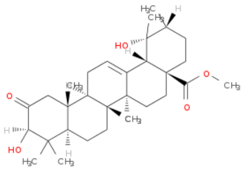	4.54	4.5	-0.04
21	T-135	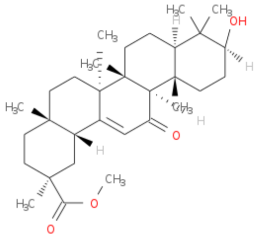	4.65	4.6	-0.05

22	T-158	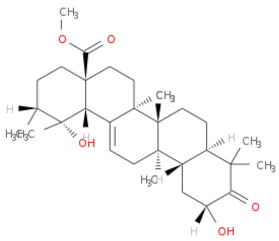	5.03	4.8	-0.23
23	T-692	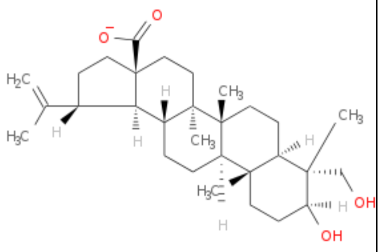	4.33	4.4	0.07
24	T-815	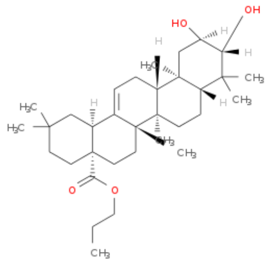	4.87	4.8	-0.07
25	T-219	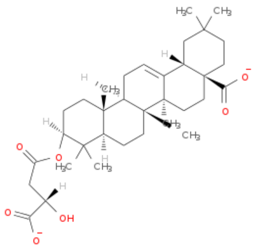	4.79	4.8	0.01
26	T-583	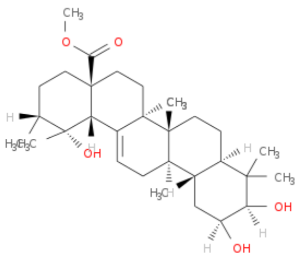	4.65	4.8	0.15

27	T-831	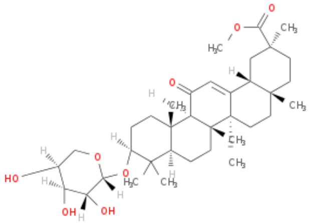	4.78	4.8	0.02
28	T-327	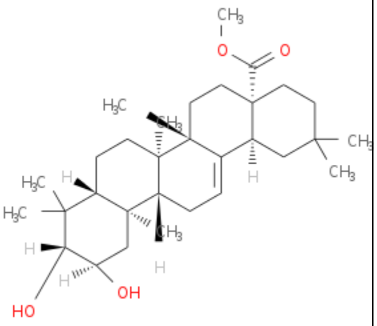	4.79	4.8	0.01
29	T-078	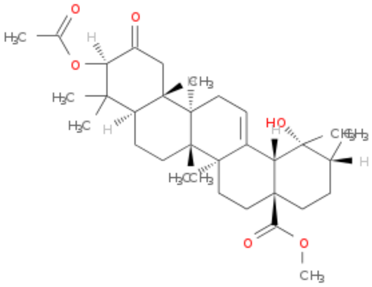	5.09	4.9	-0.19
30	T-862	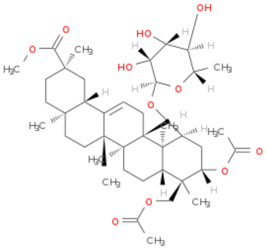	6.22	6.3	0.08
31	T-624	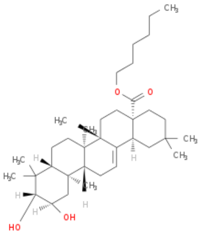	4.89	4.8	-0.09

32	T-945	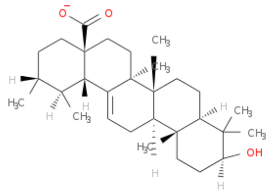	4.91	4.7	-0.21
33	T-131	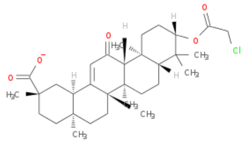	4.6	4.7	0.1
34	T-362	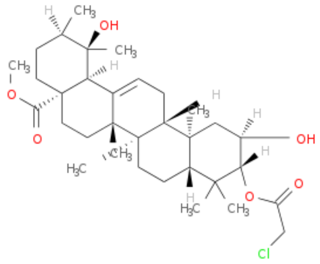	5.16	5.3	0.14
35	T-534	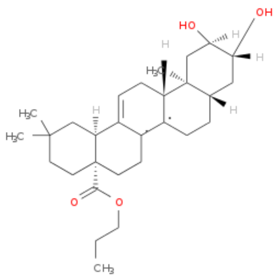	4.71	4.8	0.09
36	T-584	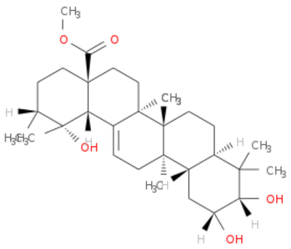	4.75	4.8	0.05

37	T-715	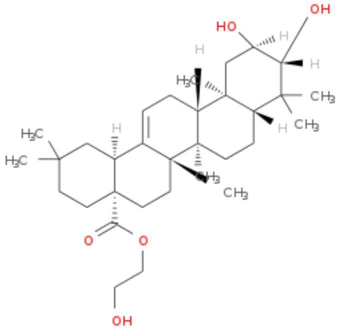	4.62	4.7	0.08
38	T-264	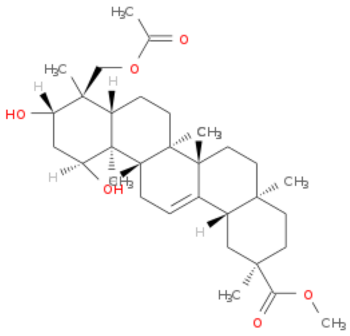	5.34	5.5	0.16
39	T-417	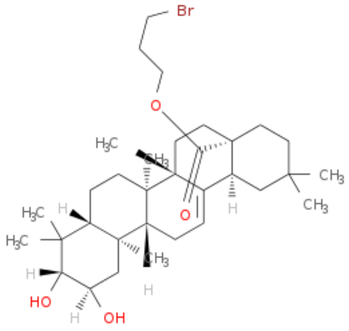	4.68	4.8	0.12
40	T-134	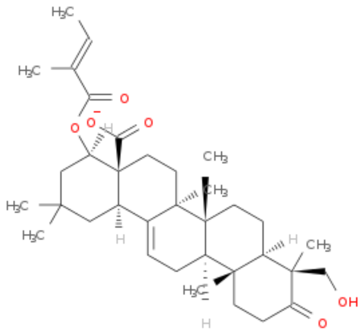	4.95	4.7	-0.25

41	T-220	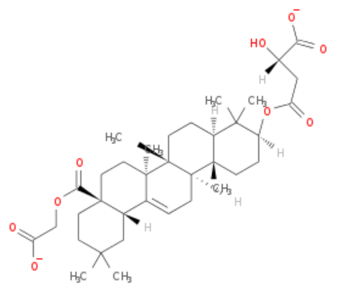	4.45	4.5	0.05
42	T-015	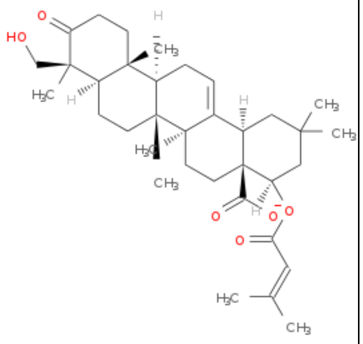	4.37	4.4	0.03
43	T-535	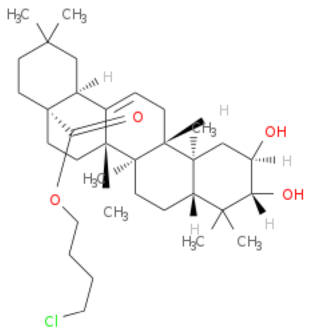	5.07	4.9	-0.17
44	T-265	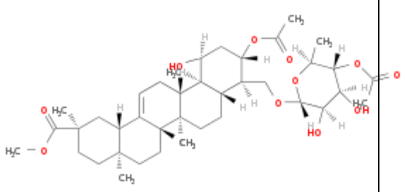	6.22	6.2	-0.02

45	T-193	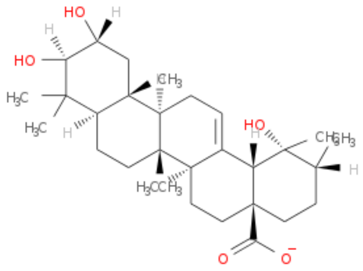	4.49	4.6	0.11
46	T-286	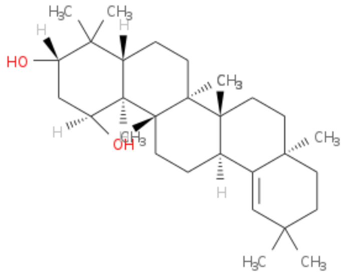	5.41	5.4	-0.01
47	T-257	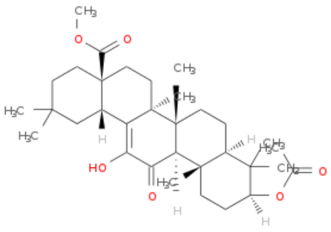	5.03	5	-0.03

Footnote: *Measured and predicted value in pIC_{50}; **the difference between predicted and experimental activity values represented as error factor, with a negative sign, if the actual activity higher than predicted activity. **Abbreviations:** pIC_{50}, negative of the log inhibitory concentration to 50% of the population.

Table S2. Details of the test set compounds with experimental and predicted anticancer activities.

Serial No.	Compound		Experimental activity (pIC_{50})*	Predicted activity (pIC_{50})*	Error factor**

1	T-804	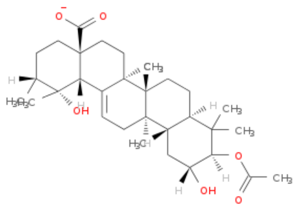	4.58	4.9	0.32
2	T-622	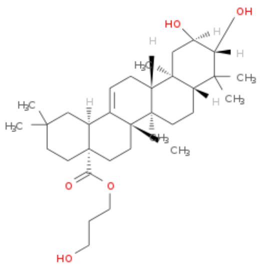	4.44	4.9	0.46
3	T-728	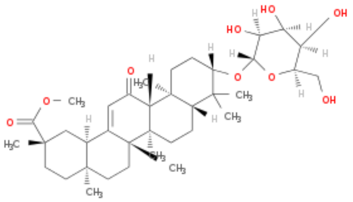	4.7	5	0.3
4	T-922	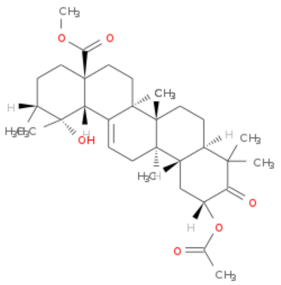	4.83	5.3	0.47
5	T-970	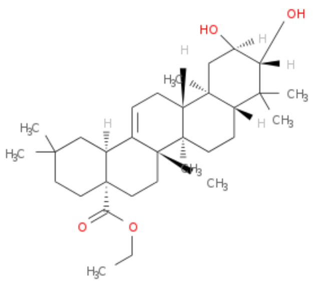	5.06	4.9	-0.16

6	T-861	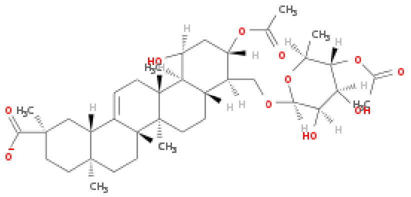	5.6	5.7	0.1
7	T-501	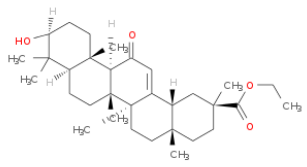	4.73	4.6	-0.13
8	T-972	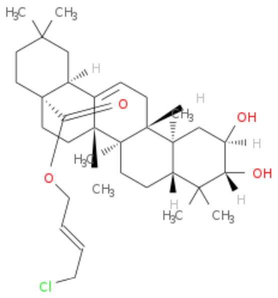	4.63	4.8	0.17
9	T-015	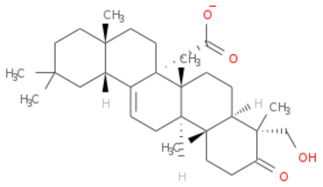	4.41	5.2	0.79
10	T-543	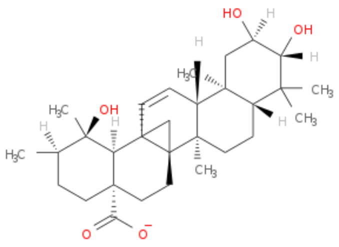	5.44	4.5	-0.94

11	T-285	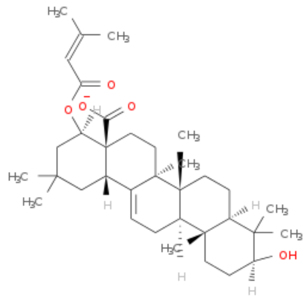	5.28	4.7	-0.58
12	T-132	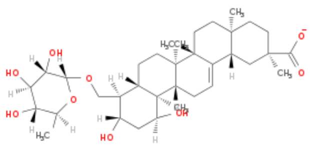	4.61	5.4	0.79
13	T-133	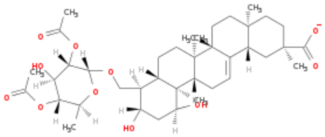	5.04	5.6	0.56
14	T-014	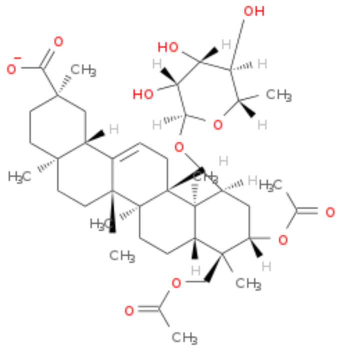	4.98	6.2	1.22

15	T-8131	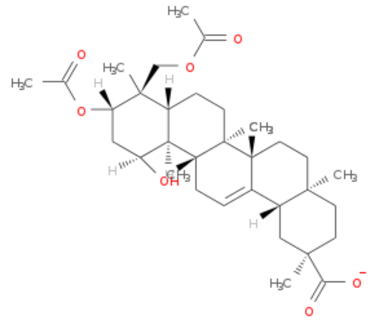	5.22	5.6	0.38
16	T-727	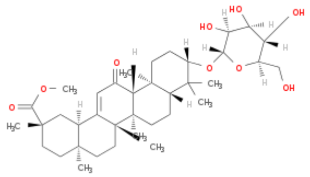	5.02	4.6	-0.42
17	T-760	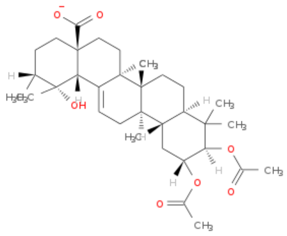	4.69	5.1	0.41
18	T-977	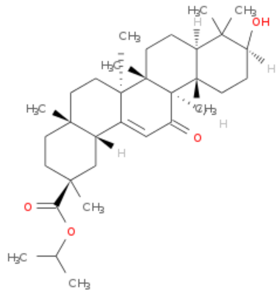	4.79	4.6	-0.19
19	T-803	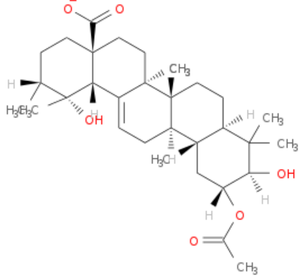	4.6	4.9	0.3

20	T-971	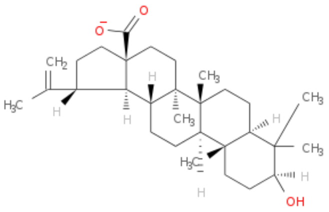	4.91	4.6	-0.31
21	T-673	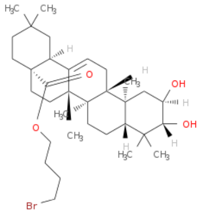	4.89	4.8	-0.09
22	T-617	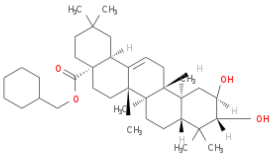	5.09	4.8	-0.29
23	T-859	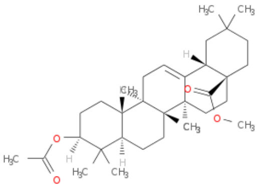	4.78	5.3	0.52
24	T-516	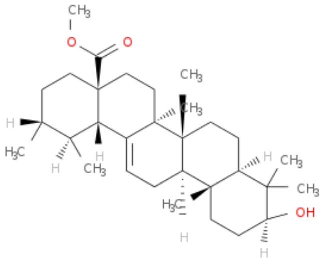	4.65	4.9	0.25

25	T-049	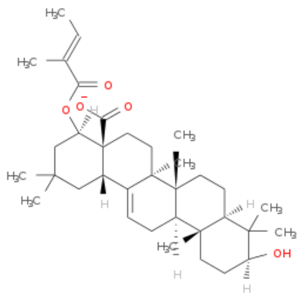	5.22	4.8	-0.42
26	T-533	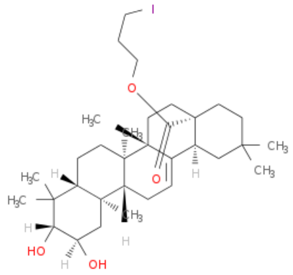	4.87	4.8	-0.07
27	T-695	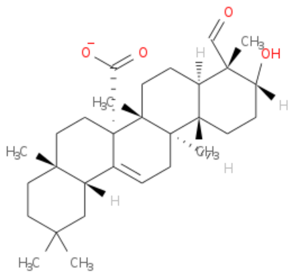	4.33	5.3	0.97

Footnote: *Measured and predicted value in pIC_{50}; **the difference between predicted and experimental activity values represented as error factor, with a negative sign, if the actual activity higher than predicted activity. **Abbreviations:** pIC_{50}, negative of the log inhibitory concentration to 50% of the population.

Table S3. Details of predicted set compounds with predicted anticancer activity, distance to model estimation, ADMET score and predicted ADMET risk parameters.

Serial No.	Compound name	Predicted activity (pIC_{50})	Distance to model	ADMET score	ADMET risk parameters
1.	P-645	4.6	Good	2.76	Size,ch,Kow,Sw,rat,3A4
2.	P-332	4.7	OK	3.96	Size,ch,Kow,Peff,rat
3.	P-630	4.9	Good	3.96	Size,ch,Kow,rat,3A4
4.	P-264	4.6	Good	3.97	Size,ch,Kow,Peff,rat
5.	P-175	4.3	Excellent	3.99	Size,ch,Kow,Peff,rat,3A4
6.	P-855	4.5	OK	4.12	Size,ch,Kow,Sw,rat,3A4
7.	P-031	4.2	OK	4.22	Size,ch,Kow,Sw,rat,3A4
8.	P-902	5.2	OK	4.22	Size,ch,Kow,Sw,rat

9.	P-701	5.2	OK	4.28	Size,ch,Kow,Peff,rat,2C9,3A4
10.	P-312	5	OK	4.41	Size,ch,Kow,Peff,rat
11.	P-252	4.7	OK	4.44	Size,ch,Kow,Peff,rat
12.	P-359	4.8	OK	4.45	Size,ch,Kow,Sw,rat,3A4
13.	P-557	4.6	OK	4.49	Size,ch,Kow,Sw,rat,3A4
14.	P-724	4.8	OK	4.51	Size,HD,ch,Peff,rat,Hp
15.	P-959	4.6	Good	4.54	Size,ch,Kow,Peff,rat,3A4
16.	P-192	4.7	OK	4.62	Size,ch,Kow,Sw,rat,3A4
17.	P-508	4.9	Excellent	4.87	Size,ch,Kow,Sw,rat,2C9,3A4
18.	P-921	4.8	OK	4.94	Size,ch,Kow,Sw,rat,3A4
19.	P-825	4.8	Excellent	4.96	Size,ch,Kow,Peff,rat
20.	P-240	4.8	Good	5.00	Size,ch,Peff,rat,Hp
21.	P-306	5.2	Good	5.15	Size,ch,Kow,Sw,rat,2C9,3A4
22.	P-464	5	Good	5.17	Size,ch,Kow,Sw,fu,rat,3A4
23.	P-984	4.9	Good	5.17	Size,ch,Kow,Sw,rat,2C9,3A4
24.	P-211	4.5	Excellent	5.20	Size,ch,Kow,Sw,rat,2C9,3A4
25.	P-673	4.7	OK	5.20	Size,ch,Kow,Peff,rat,mi
26.	P-314	4.9	Good	5.22	Size,ch,Kow,Sw,rat,3A4
27.	P-654	5	Good	5.28	Size,ch,Kow,Peff,rat,Hp
28.	P-708	4.8	OK	5.30	Size,ch,Kow,Peff,rat,3A4,mi
29.	P-526	4.8	Good	5.50	Size,ch,Kow,Sw,rat,Hp,3A4
30.	P-422	4.9	OK	5.52	Size,ch,Kow,Sw,rat,2C9,3A4
31.	P-3845	4.8	OK	5.73	Size,ch,Kow,Sw,fu,rat,3A4
32.	P-027	4.4	OK	5.73	Size,HD,ch,Peff,rat,Hp
33.	P-227	4.6	OK	5.90	Size,ch,Kow,Sw,rat,Hp,3A4
34.	P-579	4.5	Excellent	5.99	Size,HD,ch,Peff,rat,Hp
35.	P-878	4.9	Good	6.00	Size,HD,ch,Peff,rat,Hp
36.	P-668	4.7	OK	6.33	Size,ch,Kow,Sw,fu,rat,2C9,3A4
37.	P-932	5	Good	6.49	Size,ch,Kow,Peff,Sw,rat,Hp,mi
38.	P-943	4.7	OK	6.49	Size,ch,Kow,Sw,fu,rat,3A4
39.	P-200	4.8	OK	6.73	Size,ch,Kow,Sw,fu,rat,3A4
40.	P-454	4.9	OK	6.97	Size,ch,Kow,Sw,fu,rat,2C9,3A4,CL
41.	P-937	4.8	Good	7.16	Size,ch,Kow,Sw,fu,rat,2C9,3A4,CL

Table S4: Comparative evaluation of calculated bio-physicochemical properties of compound P-902 (maslinic acid analog) and anticancer drug topotecan (control).

**Properties**	**Compound P-902**	**Topotecan**
S+Acidic_pKa	5.22	9.73
S+Mixed_pKa	None	None
S+Basic_pKa	None	7.57; 2.49
DiffCoef	0.539	0.65
MlogP	4.753	1.6

S+logP	5.858	1.9
S+logD	3.695	1.51
S+logHLC	-8.735	-12.51
S+Peff	3.686	0.97
S+MDCK	50.684	190.26
S+Pcornea	224.475	78.05
S+Pskin	113.623	1.9
S+Sw	0.008	4.27E-01
S+pH_Satd	5.118	8.64
S+S_Intrins	0.005	3.66E-01
S+SF	1932.962	1.41E+02
S+S_pH	0.693	9.06E-01
S+FaSSGF	4.015E-4	1.73E+00
S+FaSSIF	0.079	2.34E-01
S+FeSSIF	0.201	3.83E-01
S+SSR	SupSat	SupSat
S+BBB_Filter	Low	Low
S+LogBB	-0.649	-0.43
S+PrUnbnd	1.657	17.45
S+Vd	0.477	1.85
S+RBP	0.565	1.04
S+fumic	0.264	0.735
S+Pgp_Substr	No (75%)	Yes (65%)
S+Pgp_Inh	No (60%)	No (94%)
S+OATP1B1_Inh	Yes (80%)	No (97%)
S+HIVI-ST	5.050	4.34
S+HIVI-TC	4.864	4.3
S+Absn_Risk	3.26	0
S+Absn_Code	Size,ch,Kow,Sw	
RuleOf5	1	0
RuleOf5_Code	LP	

Table S5. Comparative evaluation of metabolism parameters for compound P-902 (maslinic acid analog) and anticancer drug topotecan (control).

Properties	Compound P-902	Topotecan
MET_1A2_Inh	No (97%)	No
CYP_1A2_Substr	Yes (78%)	No
CYP_1A2_Sites	NonSubstrate	NonSubstrate
MET_1A2_Km	NonSubstrate	NonSubstrate
MET_1A2_Vmax	NonSubstrate	NonSubstrate
MET_1A2_CLint	NonSubstrate	NonSubstrate
CYP_2A6_Substr	No	No
CYP_2A6_Sites	NonSubstrate	NonSubstrate
CYP_2B6_Substr	No	No
CYP_2B6_Sites	NonSubstrate	NonSubstrate
CYP_2C8_Substr	Yes (58%)	No
CYP_2C8_Sites	C33(897); C20(869); C1(642);	NonSubstrate
MET_2C9_Inh	Yes (58%)	No
CYP_2C9_Substr	No	No
CYP_2C9_Sites	NonSubstrate	NonSubstrate
MET_2C9_Km	NonSubstrate	NonSubstrate
MET_2C9_Vmax	NonSubstrate	NonSubstrate

MET_2C9_CLint	NonSubstrate	NonSubstrate
MET_2C19_Inh	No	No
CYP_2C19_Substr	No	No
CYP_2C19_Sites	NonSubstrate	NonSubstrate
MET_2C19_Km	NonSubstrate	NonSubstrate
MET_2C19_Vmax	NonSubstrate	NonSubstrate
MET_2C19_CLint	NonSubstrate	NonSubstrate
MET_2D6_Inh	Yes	Yes
CYP_2D6_Substr	Yes	Yes
CYP_2D6_Sites	NonSubstrate	C31(991); C30(991); C19(744); C26(707)
MET_2D6_Km	NonSubstrate	1.32E+01
MET_2D6_Vmax	NonSubstrate	8.37E+00
MET_2D6_CLint	NonSubstrate	5.07E+00
CYP_2E1_Substr	No	No
CYP_2E1_Sites	NonSubstrate	NonSubstrate
MET_3A4_Inh	yes	Yes
CYP_3A4_Substr	yes	Yes
CYP_3A4_Sites	C33(768); C3(678); C20(551); C1(545), C18(478)	C31(998); C30(998); C26(897); C19(745)
MET_3A4_Km	51.402	5.74E+01
MET_3A4_Vmax	6.456	1.80E+00
MET_3A4_CLint	13.940	3.48E+00
MET_3A4_HLM_Km	51.168	5.97E+01
MET_3A4_HLM_Vmax	2.344	8.04E-01
MET_3A4_HLM_CLint	45.813	1.35E+01
MET_HLM_Total_CLint	11.051	1.64E+01
MET_3A4_I_mid	Yes	Yes
MET_3A4_I_tes	No	No
MET_3A4_Ki_mid	46.028	14.938
MET_3A4_Ki_tes	9.465	61.559
MET_UGT1A1	No	Yes
MET_UGT1A3	yes	No
MET_UGT1A4	No	No
MET_UGT1A6	No	No
MET_UGT1A8	No	Yes
MET_UGT1A9	No	No
MET_UGT1A10	No	Yes
MET_UGT2B7	yes	No
MET_UGT2B15	No	Yes
CYP_Risk	0.00	0

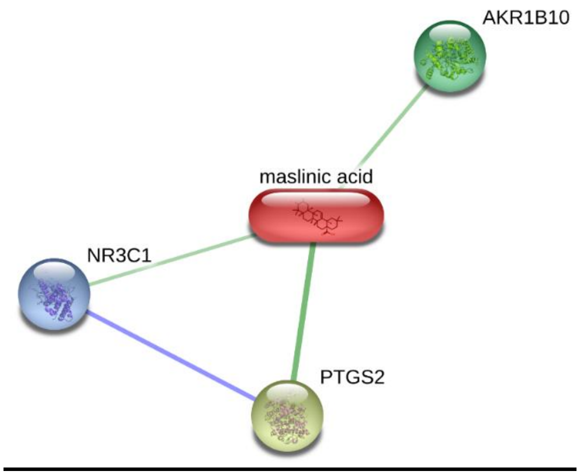

Figure S1: Representation of predicted molecular interaction network for maslinic acid and interacted target proteins.

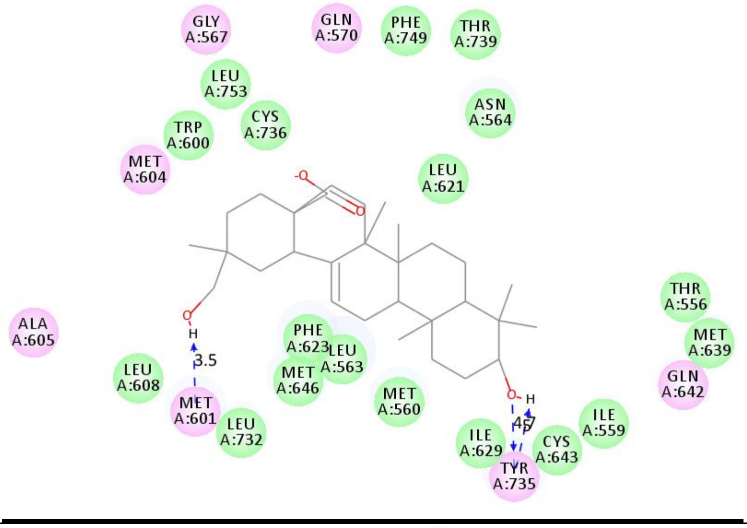

P-902

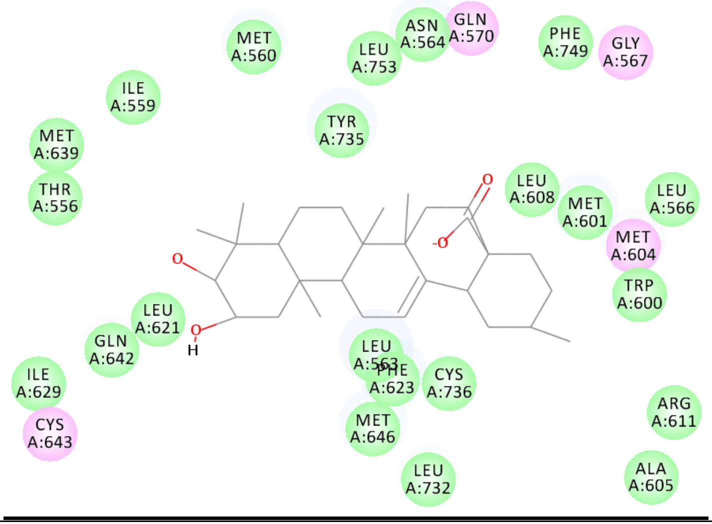

P-701

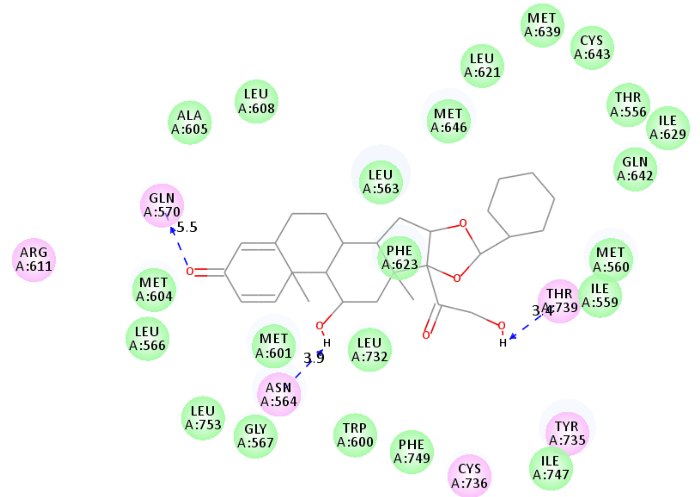	Cv7

Figure S2: The 2D diagrams illustrating protein–ligand interactions of compound P-902, P-607 and CV7 (co-crystalized inhibitor of human anticancer target NR3C1; PDB: 1ZXM).
